# Internet-Based Implementation of Non-Pharmacological Interventions of the "People Getting a Grip on Arthritis" Educational Program: An International Online Knowledge Translation Randomized Controlled Trial Design Protocol

**DOI:** 10.2196/resprot.3572

**Published:** 2015-02-03

**Authors:** Lucie Brosseau, George Wells, Sydney Brooks-Lineker, Kim Bennell, Cathie Sherrington, Andrew Briggs, Daina Sturnieks, Judy King, Roanne Thomas, Mary Egan, Laurianne Loew, Gino De Angelis, Lynn Casimiro, Karine Toupin April, Sabrina Cavallo, Mary Bell, Rukhsana Ahmed, Doug Coyle, Stéphane Poitras, Christine Smith, Arlanna Pugh, Prinon Rahman

**Affiliations:** ^1^School of Rehabilitation SciencesFaculty of Health SciencesUniversity of OttawaOttawa, ONCanada; ^2^Faculty of MedicineDepartment of Epidemiology and Community MedicineUniversity of OttawaOttawa, ONCanada; ^3^The Arthritis SocietyResearch DepartmentOntario DivisionToronto, ONCanada; ^4^Center for Health, Exercise and Sports MedicineDepartment of PhysiotherapyUniversity of MelbourneMelbourneAustralia; ^5^Sydney Medical SchoolMusculoskeletal DivisionUniversity of SydneySydneyAustralia; ^6^George Institute of HealthLevel 13321 Kent StSydneyAustralia; ^7^Research, Knowledge and PolicyArthritis and OsteoporosisVictoriaAustralia; ^8^School of Physiotherapy and Exercise ScienceCurtin UniversityPerthAustralia; ^9^Neuroscience ResearchUniversity of New South WalesSydneyAustralia; ^10^Education - Academic AffairsMontfort HospitalOttawa, ONCanada; ^11^Children's Hospital of Eastern Ontario Research InstituteOttawa, ONCanada; ^12^Faculty of MedicineUniversity of TorontoToronto, ONCanada; ^13^Department of CommunicationFaculty of ArtsUniversity of OttawaOttawa, ONCanada; ^14^Department of Community Health and EpidemiologyDalhousie UniversityHalifax, ONCanada

**Keywords:** rheumatoid arthritis, technology, knowledge translation, clinical trial, social media

## Abstract

**Background:**

Rheumatoid arthritis (RA) affects 2.1% of the Australian population (1.5% males; 2.6% females), with the highest prevalence from ages 55 to over 75 years (4.4-6.1%). In Canada, RA affects approximately 0.9% of adults, and within 30 years that is expected to increase to 1.3%. With an aging population and a greater number of individuals with modifiable risk factors for chronic diseases, such as arthritis, there is an urgent need for co-care management of arthritic conditions. The increasing trend and present shifts in the health services and policy sectors suggest that digital information delivery is becoming more prominent. Therefore, it is necessary to further investigate the use of online resources for RA information delivery.

**Objective:**

The objective is to examine the effect of implementing an online program provided to patients with RA, the People Getting a Grip on Arthritis for RA (PGrip-RA) program, using information communication technologies (ie, Facebook and emails) in combination with arthritis health care professional support and electronic educational pamphlets. We believe this can serve as a useful and economical method of knowledge translation (KT).

**Methods:**

This KT randomized controlled trial will use a prospective randomized open-label blinded-endpoint design to compare four different intervention approaches of the PGrip-RA program to a control group receiving general electronic educational pamphlets self-management in RA via email. Depending on group allocation, links to the Arthritis Society PGrip-RA material will be provided either through Facebook or by email. One group will receive feedback online from trained health care professionals. The intervention period is 6 weeks. Participants will have access to the Internet-based material after the completion of the baseline questionnaires until the final follow-up questionnaire at 6 months. We will invite 396 patients from Canadian and Australian Arthritis Consumers’ Associations to participate using online recruitment.

**Results:**

This study will build on a pilot study using Facebook, which revealed promising effects of knowledge acquisition/integration of the evidence-based self-management PGrip educational program.

**Conclusions:**

The use of online techniques to disseminate knowledge provides an opportunity to reduce health care costs by facilitating self-management of people with arthritis. Study design strengths include the incorporation of randomization and allocation concealment to ensure internal validity. To avoid intergroup contamination, the Facebook group page security settings will be set to “closed”, thus allowing only invited participants to access it. Study limitations include the lack of participant blinding due to the characteristics of this KT randomized controlled trial and a potential bias of recruiting patients only online, though this was proven effective in the previous pilot study.

**Trial Registration:**

Australian New Zealand Clinical Trials Registry ACTRN12614000397617; http://www.anzctr.org.au/TrialSearch.aspx (Archived by WebCite at http://www.webcitation.org/6PrP0kQf8).

## Introduction

### Overview

With an aging population and a greater number of individuals with modifiable risk factors for chronic diseases such as arthritis, there is an urgent need for co-care management of arthritic conditions (pharmacological as well as non-pharmacological management). Given that there is no cure for rheumatoid arthritis (RA), patients need ready access to effective self-management programs to optimize their quality of life and reduce the burden on the limited number of health care professionals in both Canadian and Australian health systems, especially in rural locations [1,2]. RA affects 2.1% of the Australian population (1.5% males, 2.6% females), with the highest prevalence from 55-75 years (4.4-6.1%). By 2032, the number of Australians with RA is projected to increase by 40% to 0.7 million [3]. Rheumatoid arthritis affects approximately 0.9% of Canadian adults, and within 30 years it will increase to 1.3% [4]. RA is a significant source of disability and economic burden for individuals and health systems [3,5]. Allowing patients with RA to have easy access to effective self-management programs will increase patient self-efficacy, optimize their quality of life (QOL), and reduce the burden on the limited number of health care professionals in both Canadian and Australian health systems, especially in rural locations [1,2,6].

### Internet-Based Health Behavior Change Programs

In general, traditional face-to-face self-management patient education programs, such as didactic lecture, videotape on RA-related information, one-to-one teaching, group education (cognitive-behavioral), and booklet or workbook are effective in the management of RA [7,8]. However, innovative low-cost and effective methods for disseminating self-management programs to a large proportion of individuals are necessary given the increasing burden caused by physical inactivity and chronic disease [9,10]. One of the growing trends today in health care delivery includes online services and information delivered or enhanced through the Internet and related information communication technologies (ICT) [11,12]. Internet-delivered interventions have been shown to produce small, but significant improvements in health behavioral changes among the general adult population [13], with a potential for having powerful implications for those with arthritis [14]. Evidence shows that patients are beginning to rely on the Internet more frequently than their physicians as a source of health information; however, they still wish to discuss Internet-based health information with their health providers [15,16]. Current recommendations for future evaluation of the implementation of evidence-based self-management programs through online interventions [6,17-21] state that the following have not been adequately evaluated: cost effectiveness, the comparative effectiveness of different online knowledge translation (KT) strategies, and which components of complex interventions provide the greatest benefit.

One ICT method that has not been well explored in rehabilitation is social media, such as Facebook, Google+, Twitter, and LinkedIn. Although social media sites are attractive for disseminating public health messages, they remain underused by health care professionals despite their low cost and wide reach [16]. A recent systematic review [13] had nine of the 10 included studies considering the efficacy of interventions, such as online health social network websites (n=2), research health social network websites (n=3), and multi-component interventions delivered in part via pre-existing popular online social network websites (Facebook: n=4 and Twitter: n=1). This systematic review revealed significant improvements in outcome measures related to health behavior change (effect sizes ranging from -0.05 (95% CI -0.45 to 0.35) to 0.84 (95% CI 0.49-1.19) [13]. Facebook has also been shown to be a successful tool for recruiting and communicating with a research team, even in a multinational context [22]. It provides a readily accessible portal for patients and health care professionals to share their experiences of investigation, diagnosis, and management of disease [23]. In addition, Facebook has been the medium for a learning strategy, which included external experts and thought leaders, providing professional communication via social media [24]. Facebook has not been used to deliver an effective self-management strategy in arthritis according to existing published protocols and studies using social media and ICT as a KT strategy [21,25-33].

This complex randomized controlled trial (RCT) will identify which component of various patient education approaches delivered through different ICT methods is an important catalyst for stronger effect sizes and sustainable results compared to the control condition.

**Figure 1:**
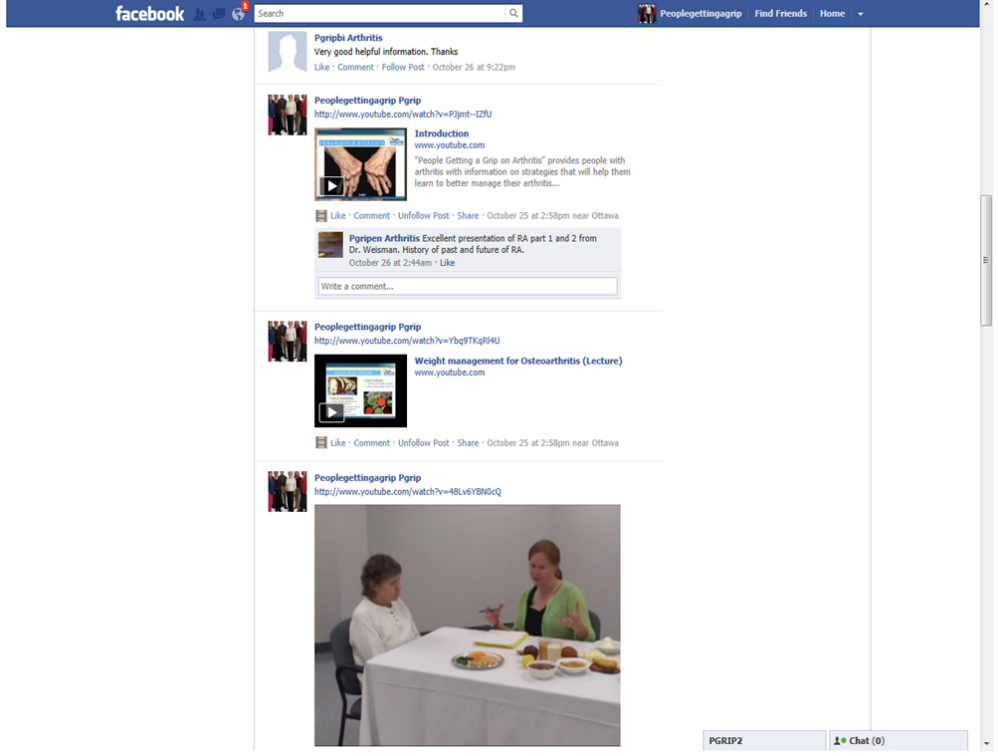
Screen caption of the Facebook group page for People Getting a Grip (PGrip) on arthritis.

People Getting a Grip on Arthritis (PGrip) is an evidence-based educational program [34] that is based on the Ottawa Panel guidelines [34-36]. It consists of education about numerous effective non-pharmacological self-management interventions for arthritis to improve health behavioral changes such as self-efficacy [37]. PGrip has been adapted by primary care providers and translated into lay words for patients to improve arthritis care in the community. The proposed PGrip-RA program will provide updated material. For the purpose of the proposed study, the PGrip program will be made available to participants via a direct Uniform Resource Locator (URL) link to the Facebook webpage (see Figure 1) and/or The Arthritis Society (TAS) PGrip webpage via email [38].

The proposed RCT will examine the effectiveness of Facebook as a KT strategy to deliver effective self-management interventions (with or without the participation of health care professionals). The protocol builds on a pilot project by proposing a larger-scale RCT that involves health care professionals and electronic dissemination of the self-management guidelines and broadening the study to include an international site.

### Hypothesis and Objective

The general hypothesis is that an online program provided to patients with RA using Facebook in combination with arthritis health care professional support and electronic educational pamphlets can serve as a useful and economical method for KT. The primary research questions presented in Textbox 1 will be addressed to explore the effect of each component of the multifaceted complex KT intervention. The secondary questions are shown in Textbox 2.

**Primary clinical and KT research questions.**

Primary clinical research questions

**Figure 2:**
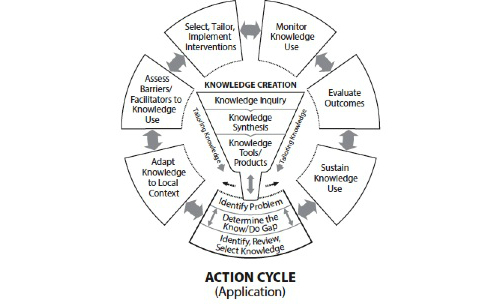
The Knowledge-To-Action cycle and study processes. Permission to use granted by Wiley oBooks (license number: 3340791020769).

**Figure 3:**
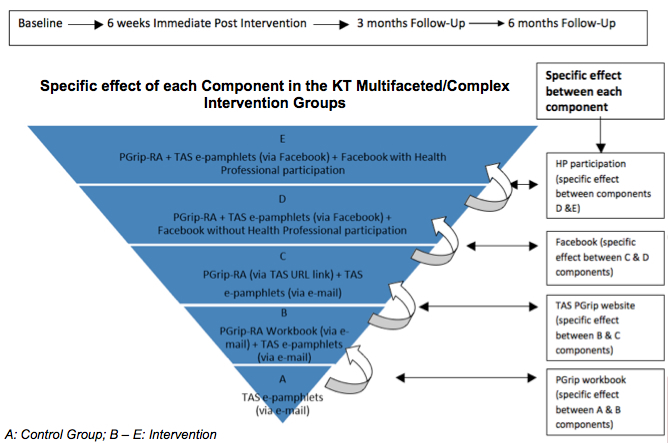
Specific effect of each component in the Knowledge Translation multifaceted/complex intervention groups.

1. Is “PGrip-RA TAS website URL link via Facebook Plus” (Group E) more effective for self-managing pain (first dimension: clinical effect) compared with the control (Group A: general electronic educational pamphlets only [No PGrip] via e-mail [No Facebook]) at 6-month follow-up (Figures 2 & 3)?
2. Is “PGrip-RA TAS website URL link via Facebook” (Group D) more effective for self-managing pain (first dimension: clinical effect) compared with the control (Group A) at 6-month follow-up (Figures 2 & 3)?
3. Is “PGrip-RA TAS website URL link via email” (Group C) more effective for self-managing pain (first dimension: clinical effect) compared with the control (Group A) at 6-month follow-up (Figures 2 & 3)?
4. Is “PGrip-RA workbook via email” (Group B) more effective for self-managing pain (first dimension: clinical effect) compared with the control (Group A) at 6-month follow-up (Figures 2 & 3)?

Primary KT research questions

1. Is “PGrip-RA TAS website URL link via Facebook Plus” (Group E) more usable (second dimension: technology/ICT effect) compared with the control (Group A) at 6-month follow-up (Figures 2 & 3)?

2. Is “PGrip-RA TAS website URL link via Facebook” (Group D) more usable (second dimension: technology/ICT effect) compared with the control (Group A) at 6-month follow-up (Figures 2 & 3)?

3. Is “PGrip-RA TAS website URL link via email” (Group C) more usable (second dimension: technology/ICT effect) compared with the control (Group A) at 6-month follow-up (Figures 2 & 3)?

4. Is “PGrip-RA workbook via email” (Group B) more usable (second dimension: technology/ICT effect) compared with the control (Group A) at 6-month follow-up (Figures 2 & 3)?

**Secondary research questions.**

A. Secondary clinical, economic, and KT outcome measures:

The group compared for the primary outcomes (Textbox 1) will also be assessed for the secondary outcomes at 6-month follow-up.

B. Improvement in outcome measures:

Changes in all primary and secondary outcomes will be assessed over time (baseline, 6 weeks, 3 months, and 6 months) for each study group comparison (Textbox 1).

C. Comparison of specific treatment study groups:

All primary and secondary outcome measures at 6-month follow-up and changes over time (baseline, 6 weeks, 3 months, and 6 months) will be compared between study groups as follows:

1. “PGrip-RA TAS website URL link via Facebook Plus” (Group E) compared with “PGrip-RA TAS website URL link via Facebook” (Group D).
2. “PGrip-RA TAS website URL link via Facebook” (Group D) compared with “PGrip-RA TAS website URL link via email” (Group C).
3. “PGrip-RA TAS website URL link via email” (Group C) compared with “PGrip-RA workbook via email” (Group B).
4. “PGrip-RA workbook via email” (Group B) compared with the control (Group A).

### Methods

This study will be guided by the milestones of the Knowledge-To-Action (KTA) framework (Figure 2) [39]. The objective of the first dimension is to examine the effect of the implementation of the PGrip-RA program on clinical and economic outcomes (ie, clinical and economic effects). The objective of the second dimension is to examine the effect of the usability of ICT (ie, Facebook and emails) as KT strategies to implement the evidence-based PGrip-RA self-management educational program (ie, technology/ICT effect).

### Study Design

The methodology used in the proposed study is in concordance with the CONSORT-EHEALTH checklist [40] (see Multimedia Appendix 1). We plan to perform an RCT that will assess five different intervention groups each receiving the PGrip-RA program delivered by different methods (Figure 3). The total intervention period is 6 weeks. Participants will have access to the online material after the completion of the baseline questionnaires up until the final follow-up questionnaire at 6 months.

This KT RCT will use a prospective randomized open-label blinded-endpoint (PROBE) design [41]. The PROBE design was selected given the nature of the study, which means the interventions, the participants, and the research coordinator administering the program will be unblinded. A blinded independent assessor will be trained to assess the online self-reported questionnaires given at the baseline, 6-week post-intervention, and at 3-month and 6-month follow-up to reduce detection bias. Investigators will be blinded to intervention assignment throughout the study period. With training and standard operating procedures, it is anticipated that any performance bias due to unblinding will be minimized. In addition, the study will use a complex intervention design, as we will be using a multifaceted intervention consisting of several educational components. In order to evaluate the effectiveness of the complex intervention, the Medical Research Council (MRC) methodological framework will be used [42]. The following key elements from the MRC framework have been accomplished: development through PGrip based on the Ottawa Panel guidelines; and feasibility and piloting, with the conduction of a previous pilot study. The evaluation and implementation elements must be completed in the proposed RCT (Figure 2); all elements will be guided by the KTA framework [39,42].

### Recruitment

#### Overview

Using online methods, we plan to recruit 396 people with RA from Arthritis Consumers’ Associations across Australia and Canada. Recruitment methods include an advertisement on the Facebook page of the Arthritis Associations (eg, TAS, Arthritis and Arthritis Consumers’ Associations’ electronic newsletter websites and other health-related websites. Potential participants will register to a universal email address and will be invited to complete an online eligibility/admission questionnaire to ensure that they meet the study’s selection criteria prior to randomization. The admission questionnaire includes information on demographics, comorbidities, comorbidities, medication intake, years of experience with ICT, expressed preference for ICT, and self-reported RA [14]. An online invitation letter with informed consent will be sent to the eligible study participants by email. Once informed consent is obtained at pre-admission, participants will be invited to complete an online baseline questionnaire. This recruitment method was approved by the University of Ottawa research ethics board for a previous pilot project using Facebook [14,43]. The recruitment process was shown to be successful in this pilot as approximately 100 participants were recruited in just over 1 month.

#### Feasibility

We anticipate similar compliance rates as in our pilot study [14]. Only 1% (1/97) of participants did not complete the baseline questionnaire, and 20% (17/97) did not complete the final 3-month follow-up questionnaire. Based on data from our pilot study [14], only 2 participants out of 99 dropped out of the study. Participants were considered dropouts if they indicated that they no longer wanted to participate in the study. The sample size has been adjusted accordingly for the proposed study.

#### Inclusion Criteria

Participants must fulfill the following criteria: (1) between 18-75 years old, (2) diagnosed with RA, (3) reside in Canada or Australia, (4) no serious comorbidities or chronic disease (eg, cancer or other illness) judged by the patient or study physician to make participation in this study inadvisable, (5) use RA-specific medications that are not expected to change during the study period, (6) self-report as inactive (30 minutes of moderate physical activity, 5 times or less per week) or not using physical interventions or agents other than prescribed medication, (7) no concurrent face-to-face consultation with a health care provider other than general practitioners or rheumatologists for RA for the recruitment period and the duration of the study, (8) capable of using and accessing the Internet weekly and a functioning email account during the study duration (6 months) (no Facebook account required, since a Facebook group page will be created specifically for this RCT), (9) free from contraindications to exercise without supervision established by the revised version of the physical activity (PA) readiness questionnaire [44], (10) able to communicate in English, (11) be a new participant (ie, not having participated in either of the two previous PGrip pilot studies), and (12) willing to sign informed consent.

#### Participant Allocation

Participants will be randomly assigned PGrip-RA to one of the two Facebook intervention groups (Groups E and D), via email only groups (Groups C and B), or the No PGrip control group (Group A) based on a sequence of computer-generated random numbers using a blocking factor (randomly varying between 4 and 6). After the potential participant registers online the PGrip Gmail account, they will be contacted by the research coordinator and their eligibility confirmed. If eligible and consenting, the participant will then be randomly allocated to one of the five study groups (Group A, B, C, D, or E) using the central randomization scheme. The research assistant, who is not involved in data collection, will contact the research study Methods Center data manager. Prior to running the randomization program, the data manager will document the participant’s initials (first and last) and date of birth (month and year). After running the program, the data manager will document the intervention assignment with the participant information, assign a study identification number (ID) and then inform the research assistant of the assignment and participant ID. This process will help ensure concealment of allocation. After randomization, the participant will be informed through email of their group assignment. Participants in the interventions groups (Groups E and D) will receive specific confidential information for login purposes.

### Intervention

#### Overview

There will be five study groups (Figure 3) in the proposed complex RCT. The PGrip evidence-based self-management educational program intervention will be provided online (via email or Facebook) for 6 weeks (Table 1). More details about intervention and control conditions are provided using the TIDieR checklist and guide [45] in the trial registry version. Similar online methods were used in the previous pilot study [14]. This study was approved by the University of Ottawa Ethics Committee (certificate number: H11-12-10).

#### PGrip-RA TAS Website Link via Facebook Plus (Group E)

Participants in the Facebook Plus group (Group E) will have access to a Facebook group page, which will present the PGrip-RA online program. Using the material from the previous pilot study [14], the PGrip-RA online Facebook page will include YouTube video presentations of various effective RA self-management intervention strategies based on the Ottawa Panel guidelines [34,35]. Similar to the PGrip pilot study [14,43], YouTube videos will include narrated PowerPoint presentations with simplified, concise instructions on how to perform/apply each self-management intervention and case studies illustrating their appropriateness and relevance. In addition, YouTube video presentations of practical sessions including a health care professional describing step-by-step instructions while performing the evidence-based intervention will also be posted on each Facebook group page. Participants will have the opportunity to share their unique perspective on living with arthritis and how they plan to integrate the effective self-management interventions into their daily lives by posting comments on the “wall” of the Facebook group page. Participants will take part in three separate self-management online modules, each over the course of 2 weeks, consisting of (1) physical activity interventions, (2) wrist orthotics and foot insoles interventions, and (3) transcutaneous electrical nerve stimulation (TENS) interventions. A group of three trained health care professionals with at least 1 year of clinical experience with individuals with RA will represent three professions (physiotherapy, occupational therapy, and kinesiology). An advertisement will be posted on the Arthritis Health Profession Association (AHPA) website. An interview will be performed based on their clinical experience, expertise, and ICT abilities. A general orientation on the nature and relevance of these three effective interventions will be provided. They will also be asked to read the comments and questions that participants write to each other on the “wall” and will give feedback to the participants on a weekly basis to fulfill the participants’ needs (Table 1).

**Table 1:** Facebook Plus (Group E) module including health care professionals.

Module (6 weeks total)	Moderator
Physical activity (PA) interventions (2 weeks)	Physiotherapist #1 and kinesiologist #1 (English)
Wrist orthotics and foot insoles interventions (2 weeks)	Occupational therapist #1 (English)
TENS interventions (2 weeks)	Physiotherapist #1 (same as PA) (English)

The health care professionals will participate in a half-day workshop at the University of Ottawa prior to the study, where they will receive training and information on evidence-based practice and the selected self-management interventions [46-52]. Training will consist of Ottawa Panel guidelines, PGrip-RA material using PowerPoint presentations and videos, and frequently asked questions from the pilot study [14]. One physiotherapist and one kinesiologist will be responsible for the physical activity module. An occupational therapist will cover the module with wrist orthotics and foot insoles. The same physiotherapist will also cover the TENS module. During each 2-week module, the respective health care professional(s) will monitor the Facebook page on three separate days (Monday, Wednesday, and Friday for 4 hours each day), review all of the participants’ written comments, and provide feedback (Figure 3). Health care professionals involved in Group E will help Group E participants set goals for self-management interventions offered in PGrip-RA. Goal setting will not be required for the participants in the four other groups. However, study participants in Group E will record their physical activities and participation in PGrip interventions using the 7-day Physical Activity Readiness (PAR) calendar [53] included in electronic logbooks (e-logbooks) during the 6 weeks of the intervention and at 3-month and 6-month follow-up. Goal attainment and intervention adherence will be measured in Group E by comparing individual records with what is recommended for each intervention in the PGrip program.

AHPA has agreed to recruit health care professionals with expertise in arthritis/RA on their website and newsletters. In addition, participants will be provided with TAS educational pamphlets on self-management interventions for RA (general information) by posting URL links for each on the Facebook page. The TAS educational e-pamphlets on general self-management interventions for RA will include (1) Rheumatoid Arthritis: Know your options [54] and (2) Physical Activity & Arthritis [55].

#### PGrip-RA TAS Website Link via Facebook (Group D)

Similar to Group E, participants in the Facebook group (Group D) will have access to a Facebook group page (separate from Group E, without the participation of health care professionals) and will participate in the three self-management modules. All participants in Group D will also be provided with TAS educational pamphlets on general self-management interventions for RA by posting a *URL* link for each on the Facebook page.

#### PGrip-RA TAS Website Link via Email (No Facebook) (Group C)

A third online intervention group (Group C) will consist of individuals being emailed (once for the entire duration of the study) a URL link to access the TAS PGrip-RA website. This website will contain the same educational information that will be provided in the Facebook groups. Individuals in this group will not have access to the two Facebook group pages and will not interact with each other or the health care professionals through written messages. Participants will also be provided with TAS educational pamphlets on general self-management interventions for RA.

#### PGrip-RA Workbook via Email (No Facebook) (Group B)

A fourth group will be emailed (once for the entire duration of the study) a workbook of similar quality with the content of the online PGrip-RA program in a Portable Document Format (PDF) file and the URL links of the electronic TAS educational pamphlets on general self-management interventions for RA. They will not have any access to the health care professionals, any of the Facebook group pages, or the online version of PGrip-RA.

#### Control With TAS Electronic Educational Pamphlets Only (No PGrip-RA) via Email (No Facebook) (Group A)

In order to avoid intergroup contamination, participants in the control group will only be emailed (once for the entire duration of the study) the URL links of the electronic TAS educational pamphlets on general self-management interventions for RA. They will not have any access to the health care professionals, the PGrip-RA material (online or PDF workbook), or any of the Facebook group pages.

### Outcome Measures

#### Overview

The outcome measures will be measured immediately after the PGrip intervention (6 weeks) and at 3-month and 6-month follow-up to determine when the intervention becomes effective and whether effects are maintained (retention effect) (Tables 2-4 and Figure 3).

A 6-month follow-up will be considered as the primary endpoint and is supported by previous studies [25,37,56] that have found significant benefits for self-efficacy of an online, as well as a face-to-face arthritis self-management program. The PGrip evidence-based self-management educational program intervention will be provided online (email or Facebook) during the 6-week duration. This length application is justified and in concordance with existing effective arthritis self-management interventions [37,57]. We will measure immediately after the PGrip intervention and also 3 months later [58] as secondary outcome measures and to see when it becomes effective and when the effects are maintained (retention effect).

**Figure 4:**
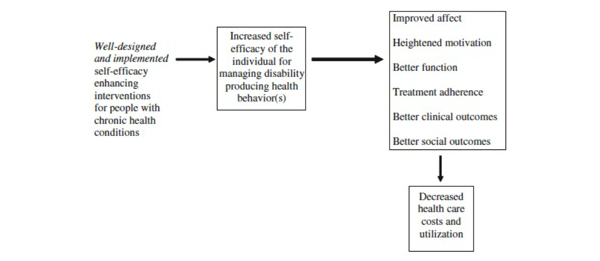
Hypothesized model of effects of self-efficacy-enhancing interventions for people with chronic diseases. Permission to use granted by SAGE Publications (license number: 3340780145743).

**Figure 5:**
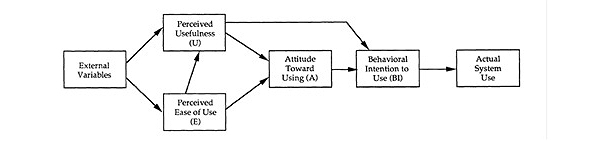
Technology Acceptance Model. Reprinted by permission. Copyright [1989] INFORMS. Fred D. Davis, Richard P. Bagozzi, Paul R. Warshaw (1989) User Acceptance of Computer Technology: A Comparison of Two Theoretical Models. Management Science 35(8):982-1003, the Institute for Operations Research and the Management Sciences, 5521 Research Park Drive, Suite 200, Catonsville, Maryland 21228, USA.

There are two theoretically based dimensions refining the KTA framework concepts for Monitoring Knowledge Use and Evaluated Outcomes (Tables 2-4; [14,53,59-71]). The first dimension is to examine the effect of the implementation of the PGrip-RA program on clinical and economic outcome measures. The Hypothesized Model of Effects of Self-Efficacy-Enhancing Interventions for People with Chronic Diseases (HMESE) (Figure 4) [59] is adapted from the Self-Efficacy and Social Cognitive Theory developed by Bandura [72] and by Lorig [60] for arthritis and chronic disease assessment purposes [60,72]. The second dimension is to examine the effect of the usability of Facebook and emails as KT strategies to implement the evidence-based PGrip-RA educational program. This will be measured by the Diffusion of Innovation Model (DIM) [61] and more specifically the Technology Acceptance Model (TAM) (Figure 5) [62].

**Table 2:** Assessment schedule and additional outcome measures.

Assessment	Admission	Baseline	6 weeks post intervention	3-month follow-up	6-month follow-up
Informed consent (pre-admission)	X				
Demographics	X				
Self-reported diagnosis of RA	X				
Physical Activity Readiness Questionnaire (PAR-Q) [44]	X				
Self-efficacy to manage pain		X	X	X	X
Prior knowledge of self-management programs (SMPs)		X			
Attained knowledge of SMPs			X	X	X
Intention to use SMPs			X		
Actual use of SMPs				X	X
Self-efficacy (function)		X	X	X	X
Quality of life (EQ-5D) [63]		X	X	X	X
Health resource utilization		X	X	X	X
Usability with online learning		X	X	X	X
Self-reported pain (visual analogue scale)		X	X	X	X
e-logbook (daily) using 7-day Physical Activity Recall (PAR) calendar (Facebook Plus /Group E only)		X	X	X	X
7-day PAR (periodic) [53]		X	X	X	X
Long-term goal attainment scaling (Facebook Plus /Group E only)			X	X	X

**Table 3:** Outcome measures according to selected measurement frameworks: KTA monitoring knowledge use.

Concept	Theory-based	Operationalization	Time period	Dimension
Knowledge acquisition (Secondary outcome measure for first dimension)	DIM [61]; TAM [62]	Questionnaire developed in pilot study	Baseline & 6 weeks	PGrip-RA: clinical dimension
Intention to use (Secondary outcome measure for first dimension)	DIM [61]; TAM [62]	Questionnaire developed in pilot study; goal setting for Facebook Plus /Group E only	6 weeks, bi-weekly during 6 weeks, 3-month and 6-month follow-up	PGrip-RA: clinical dimension; Facebook or email^a^; technology/ICT dimension

^a^Facebook or email: KT dimension using ICT.

**Table 4:** Outcome measures according to selected measurement frameworks: KTA evaluated outcomes.

Concept	Theory-based	Operationalization/ Instrumentation	Time period	Dimension
Self-efficacy (pain) (Primary outcome measure for first dimension)	DIM [61]; HMESE [59]	Arthritis self-efficacy (pain management subscale)	Baseline, 6 weeks, 3-month and 6-month follow-up	PGrip-RA: clinical dimension
Actual use (Secondary outcome measure for first dimension)	DIM [61]; TAM [62]; HMESE [59]	Questionnaire developed in previous pilot study [14]; 7-day physical activity readiness (PAR) calendar [53] and changes report [65] (periodic) for the previous week	3-month and 6-month follow-up	PGrip-RA: clinical dimension
		e-logbooks & Long-Term Goal Attainment [64] (Facebook Plus /Group E only)		
		# log in # hits; Facebook intensity scale [66]		Facebook or email^a^: Technology/ICT dimension
Better clinical outcome measures: pain, quality of life, self-efficacy (function), motivation, social (Secondary outcome measures for first dimension)	DIM [61]; HMESE [59]	Pain intensity [67]; arthritis self-efficacy (function management/other symptom subscale) [60]; Euro QoL: EQ-5D-5L [63], mobility, self-care, pain, anxiety/depression	Baseline, 6 weeks, 3-month and 6-month follow-up	PGrip-RA: clinical dimension
Interventions adherence (Secondary outcome measures for first dimension)	DIM [61]; HMESE [59]	7 day-PAR [53] (periodic) questionnaire to measure what was their typical physical activity level and other PGrip interventions just for the previous week)	Baseline, 6 weeks, 3-month and 6-month follow-up	PGrip-RA: clinical dimension
		e-logbooks (daily) & Long-Term Goal Attainment [64] (Facebook Plus /Group E only)	6 weeks, 3-month and 6-month follow-up	
Usability (Primary outcome measure for second dimension)	DIM [61]; TAM [62]	System Usability Scale [68]; adapted TAM 2 Scale [69]	Baseline, 6 weeks, 3-month and 6-month follow-up	Facebook or email^a^: technology/ICT dimension
Better health economic outcome measures: Decreased health care costs and utilization (Secondary outcome measure for first dimension)	DIM [61]; HMESE [59]	Health Resource Utilization questionnaire [70]; quality adjusted life years (QALY) [71]	Baseline, 3-month and 6-month follow-up	PGrip-RA, Facebook or email^a^: economic dimension

^a^Facebook or email: KT dimension using ICT.

#### Self-Efficacy to Manage Pain Related to KTA Evaluate Outcomes (Primary Outcome Measure: Clinical Effect, First Dimension)

Self-efficacy is one’s belief and confidence to perform a given behavior, such as exercise [56-60,72]. Self-efficacy was chosen as the primary outcome measure, as the self-management interventions consist of various activities to improve symptoms associated with RA, principally pain. The measurement of self-efficacy will therefore capture the effectiveness of all interventions regardless of the specific type of self-management strategy. The Stanford Arthritis Self-Efficacy Scale (ASES), a valid tool with an internal consistency reliability of 0.94 [60], will be used to assess participants’ self-efficacy (Tables 2 and 4). The subscales of the ASES tool (self-efficacy to improve function and other symptoms) will be used for secondary outcome measures. The internal consistency reliability of the pain scale is 0.75 with a test-retest reliability of 0.87, while the internal consistency reliability of the pain scale is 0.87 with a test-retest reliability of 0.90 [60].

Usability With ICT Related to KTA Evaluate Outcomes

Primary Outcome Measure: Technology/ICT Effect, Second Dimension

Participants in all groups will be assessed according to their level of usability with their respective ICT KT strategy (ie, Facebook or email). The System Usability Scale (SUS) instrument (Tables 2 and 4), an empirically validated tool [68], as well as the adapted technology acceptance model (TAM) 2 scale [69], will be used to measure participants’ usability perception at baseline, 6 weeks immediate post-intervention, and 3-month and 6-month follow-ups (Tables 2 and 4).

#### Knowledge Acquisition, Related to KTA Monitoring Knowledge (Secondary Outcome Measure)

Knowledge acquisition will be measured by questionnaires used in the previous pilot study [14]. Participants’ pre-program knowledge of the self-management interventions will be assessed at baseline, and post-program knowledge will be measured at 6 weeks immediate post intervention (Tables 2 and 3). Participants will be asked to complete a series of questions using a Likert scale to determine which self-management strategy options are effective for treating RA. Knowledge acquisition related to ICT use will also be performed. Examples of how “knowledge use” and “intended use” were operationalized are presented in Table 3.

#### Intention to Use the PGrip Self-Management Interventions (Secondary Outcome Measure)

Intention to use the PGrip-RA self-management interventions will be measured via questionnaires used (Tables 2 and 3) in the previous pilot study [14]. Study participants in Group E will be asked to set goals bi-weekly regarding any self-management interventions offered by PGrip-RA with the guidance of a health care professional (Group E).

#### Actual Use of the PGrip Self-Management Interventions and ICT Related to KTA Evaluate Outcomes (Secondary Outcome Measure)

Actual use of the PGrip-RA self-management interventions will be measured by questionnaires used (Tables 2 and 4) in the two pilot studies [14,73]. The number of views of the YouTube videos and the number of comments and postings (Facebook or emails) will be recorded. Furthermore, the Facebook Intensity Scale will be used to measure participants’ overall engagement in Facebook for groups E and D only [66]. PGrip-RA program adherence will be measured with the actual use questionnaire [14] and also by calculating the proportion of the number of intervention sessions performed divided by the number of sessions prescribed (eg, walking program 3 times a week as recommended in the Ottawa Panel guidelines [34,35]) and recorded in the participants’ online logbooks. A logbook used in a previous RCT [65], will be filled out daily online (e-logbook: as exploratory outcome measure) using the validated 7-day PAR calendar [53,74] during the study duration by study participants in Group E and a bi-weekly questionnaire on potential changes in PA, medication intake, habits, and adverse events. The calendar proposed by the 7-day PAR [53] incorporated in the e-logbooks (Tables 2 and 4) will be used as a self-report questionnaire to calculate the number of intervention sessions each participant will attend each week.

However, the periodic online 7-day PAR questionnaire [53] (Tables 2 and 4) will be performed by all the study participants of the five study groups (A-E) at baseline, 6 weeks post intervention, and 3-and 6-month follow-up to measure their typical physical activity level only for the previous week. The 7-day PAR will also be adapted to record prescribed numbers of application sessions of other physical interventions (eg, physical activity, TENS) to be optimally effective according to the Ottawa Panel guidelines [34,35]. Actual individual recordings in the 7-day PAR calendar will be compared with PGrip-RA intervention recommendations using the long-term goal attainment scale [64]. Long-term goal attainment scaling is a validated tool that will measure (as an exploratory outcome measure) participants’ long-term goal attainment levels (Tables 2 and 4) in Group E only. It includes five goal attainment levels: (1) -2 (much worse than expected), (2) -1 (somewhat less than expected), (3) 0 (expected level), (4) +1 (somewhat better than expected), and (5) +2 (much better than expected) [64].

#### Self-Efficacy to Improve Function Related to KTA Evaluate Outcomes (Secondary Outcome Measures)

The self-efficacy function subscale of the ASES will be used to measure participants’ self-efficacy to improve their functional status (Tables 2 and 4). The internal consistency reliability of this scale is 0.90 with a test-retest reliability of 0.85 (Tables 2 and 4) [60].

#### Quality of Life Related to KTA Evaluate Outcomes (Secondary Outcome Measure)

Quality of life will be assessed using the EuroQoL Index (EQ-5D-5L) [63]) (Tables 2 and 4). It is the most commonly used and extensively validated measure of health-related quality of life [72]. It includes five domains: (1) mobility, (2) self-care, (3) usual activities, (4) pain/discomfort, and (5) anxiety/depression. The scoring system has 5 levels: no problems, slight problems, moderate problems, severe problems, and extreme problems [72]. The EQ-5D-5L is an integral component of the economic analysis detailed later (Tables 2 and 4). QOL will be measured at baseline, 6 weeks, 3-month, and 6-month follow-up.

#### Self-Reported Pain (Secondary Outcome Measure)

Study participants’ self-reported assessment of pain intensity will be recorded at baseline, 6 weeks, 3 month, and 6 month follow-up on an online 100-millimeter (mm) visual analogue scale (Tables 2 and 4), where 0 mm represents no pain and 100 mm maximal pain (Tables 2 and 4) [67].

#### Economic Outcomes (Secondary Outcome Measures) and Analysis

These outcomes are described in the Economic Evaluation section.

### Measurement Frequency

Four different measurement sessions will be conducted throughout this RCT for each participant in all five groups (Table 2 and Figure 3). All measurements will be performed through the use of online questionnaires and will take 45 minutes to complete. Online questionnaires will be developed using an online survey tool “Fluid Survey”, which is a Canadian and confidential database. The online questionnaire is in accordance with the CHERRIES checklist [75] and will be made accessible to participants in Groups E and D on the Facebook pages using a *URL* link to access each online questionnaire on each group page. Participants in Groups C, B, and A will be emailed the same URL link to access the questionnaires. Using the “wall” on the Facebook page for Groups E and D, our research team will provide updates and reminders to all participants regarding deadlines to complete questionnaires. As an incentive and to reduce the number of participant dropouts, the participants will receive a CAN $30 gift certificate for each completed questionnaire and a personalized certificate of participation. Prior to obtaining participants’ mailing addresses, participants will be asked to give their consent to provide this personal information in order to receive the gift certificate.

### Statistical Analysis

#### Overview

Data analysis will be performed using SPSS 21 and will be conducted on an intention-to-treat basis using multiple imputation for missing data. Descriptive statistics such as proportions, means, and standard deviation will be used to summarize baseline variables across the five study groups (Groups A-E) (Figure 3). Baseline characteristics will be assessed to ensure there are no differences among the study groups.

For the primary research questions (Textbox 1), an analysis of variance (ANOVA) will be conducted to compare groups B-E to A on the primary clinical outcome measure (ie, self-efficacy to manage pain using ASES) and primary KT outcome measure (ie, usability using SUS) at 6-month follow-up. In particular, Dunnett’s multiparameter test will compare groups B-E individually to group A on the primary outcome measure. If clinically important differences in baseline variables are found, the interventions will be compared adjusting for these baseline variables using multiple regression and similar multiparameter tests will be conducted.

For the secondary outcome measures (secondary research questions A) (Textbox 2), the same analysis strategy considered for the primary outcome measures will be followed.

Furthermore, for the change over time from baseline, 6 weeks, 3 months, and 6 months for the primary and secondary outcome measures (secondary research questions B) (Textbox 2), a two-way repeated measures ANOVA will be conducted involving the within factor time (0, 6 weeks, 3 months, 6 months) and between factor (study group), following a similar strategy as outlined above for the primary outcome measures.

In order to assess the importance of the different components making up the interventions for Groups B-E (secondary research questions C) (Textbox 2), an ANOVA will be conducted and a posterior test using Tukey’s honest significance difference test will compare Group E to D, Group D to C, Group C to B, and Group B to A. This analysis will be considered for all outcomes. In addition, the outcomes will be compared from baseline to 6 weeks immediate post intervention, and 3-month and 6-month follow-up using a two-way ANOVA with the between factor as the study groups (Groups A-E) and the within factor as time (baseline, 6 weeks, 3 months, and 6 months).

The cost-effectiveness analysis is described in the economic evaluation section below. Further, the number of visits per page will be monitored using Facebook’s group page tracking tool, and qualitative information will be collected from comments and posts on the Facebook group page wall. This qualitative data will be analyzed using a generalized content analysis approach [76].

In addition to multiple imputation for missingness, general repeated measures likelihood methods will be considered when repeated observations are available, in order to provide an assessment of the robustness of the missingness estimation.

#### Sample Size Calculations

**Figure 6:**
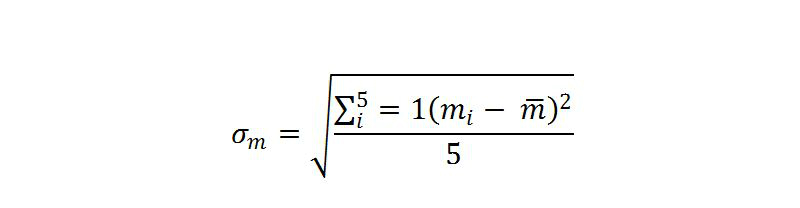
Sample size formula.

The sample size is based on the number of observations needed to compare self-efficacy to manage pain (ie, primary clinical outcome measure) and usability (primary KT outcome measure) at 6-month follow-up. In the psychometrics paper for the Stanford Arthritis Self-Management Study [60], the standard deviation of the self-efficacy to manage pain subscale of the ASES for the control group was found to be 1.79. A small effect size of 0.15 in pain self-efficacy (and similarly for usability measured by SUS [14]) was identified by the investigators as being a minimal clinically important effect size to identify. The spread in the means across the study groups is formally represented by the standard deviation of the group means (Figure 6) [77]. To detect an effect size of 0.15, the size of the variation in the means as represented by their standard deviation is 0.90, given the common standard deviation within a group measured with the self-efficacy in pain scale of the ASES of 1.79 (difference in means (.15) (1.79)=0.90). Given the self-efficacy to manage for the control group [60] of 4.82, the hypothesized means being compared for the five study groups are 4.82, 5.09, 5.36, 5.63, and 5.90. In a one-way ANOVA study, a sample size of 63 is needed for each of the five groups whose means are to be compared. The total sample of 315 subjects achieves 80% power to detect an effect size of 0.15 in the differences among the means versus the alternative of equal means using an *F* test with a 0.025 significance level (0.025 selected since there are two primary outcome measures).

With the given sample size of 63 per group, we will be able to detect an effect size of 0.21 in the SUS scale for usability. This small effect size was deemed acceptable by the study investigators. This effect size is based on a standard deviation of 3.1 from the pilot study, 80% power, 0.025 significance level, and the sample size of 63 derived for the primary clinical outcome.

To account for a potential loss to follow-up, the sample size has been adjusted to accommodate a 20% loss which is typical of the losses in similar past studies, that is, 63⁄(1-.2)=79 per group, and in total 396.

### Economic Evaluation (Secondary Outcome Measure: Economic Effect, First Dimension)

The economic analysis will be a cost-utility analysis where we will compare the costs of the five comparative groups related to their health service utilization over the 6-month period. In order to facilitate the economic analysis, estimates of total costs for each participant will be assessed at baseline and at 6-month follow-up. These will be obtained from each participant to attain an estimate over the duration of this RCT. Finally, these participant-level costs will be analyzed to obtain estimates of average costs for each of the five alternatives considered within this RCT. Estimates of resource use (over the previous 6 months) will be obtained from a health resource utilization questionnaire at baseline and 6 months included in the online questionnaires. The questionnaire will provide information on participant’s use of family physician visits, specialist visits, prescription drug use, and other related health care resource use. The questionnaire will be a modified version of one we have used in a previous study [65]. Each health and community resource will have a unit cost applied to it. The weighted sum of resource use will be used to estimate the total cost. Resource costs for hospitalization will be obtained from the Ontario Case Costing Initiative [78]. For health care professional consultation and specific procedures, costs will be obtained from the provincial fee schedule [79]. Costs for outpatient medication will be obtained from the provincial drug formulary [80]. Utility values derived from the EQ-5D-5L responses (Tables 2 and 4) will be used to estimate QALYs for the 6-month period adjusting for baseline utility. The economic analysis will compare the incremental cost per QALY gained by each intervention group (Groups B-E) compared to the control (Group A) at 6-month follow-up. In order to estimate and adjust for the uncertainty of the incremental cost and effectiveness, probabilistic analysis will be conducted using non-parametric bootstrapping [81].

## Results

This proposed RCT builds on a previous pre-post pilot study using Facebook [14], which revealed promising effects of knowledge acquisition/integration of the evidence-based self-management PGrip educational program.

## Discussion

### Strengths and Limitations

The proposed KT international study is a rigorous RCT using the PROBE design with a low-cost online intervention. The major strength of this study design is that it will use ICT to deliver information to people with RA that is both accessible and interactive. The design will be able to overcome the barriers of geographical distance between the two study sites (Canada and Australia) and resolve other disparities in care. Making use of the rapid increases in eHealth will appeal to consumers who are already consulting online sources for self-management [9,10,13,14]. Assessments will include a range of outcome measures from self-efficacy to usability to health economics. Furthermore, the study design is sustainable, easily modifiable, low-cost, and is in alignment with current primary care and chronic disease management reforms.

However, blinding participants is impossible in this type of study, as is generally the case with physical rehabilitation RCTs [82]. We recognize that the results of this study will likely be generalizable only to individuals with RA who are computer literate and have Internet access. Furthermore, we are also aware of the potential bias of recruiting patients only online, though this was efficient in the previous pilot study [14].

Self-reported diagnosis is also a limitation of this project. Since it is an online project, the investigators cannot request participants to send via email a confirmed medical diagnosis, for ethical and confidentiality issues. However, to minimize the potential misclassification bias, a specific question about confirmed diagnosis of RA will be included in the admission questionnaire delivered through the online survey tool “Fluid Survey”, which is a confidential database. This specific question will precisely describe the symptoms and criteria of RA.

Another limitation involves the timeframe of the intervention, as they will not be assessed for adherence in the long term beyond the 6-month follow-up. There is an increased risk of Type-1 error due to the presence of multiple outcomes (ie, multicollinearity).

### Challenges and Potential Solutions

The national implementation of the PGrip pilot study [14] previously identified challenges to the uptake of the best evidence for RA due to varying perceptions about facilitators and barriers in adopting effective self-management interventions for RA. These barriers will be considered by the research team developing the program. The PGrip educational program will be built into the format, delivery, and content of all online learning modules. An additional challenge will be adapting the hands-on portions of the program (interactions with patients and faculty, exercise demonstrations, assistive devices demonstrations) to an online environment. Videos will be one strategy used to address these issues as well as linking participants with local resources to provide another means of reinforcing the learning. Creating a peer support network might be another approach. These strategies will be considered by the team in the planning process, since the members provide expertise in this area.

Another challenge is with the recruitment of participants and convincing them that using Facebook will be secure. This could be solved by providing a statement on the informed consent form indicating that their information will remain confidential. Since this will be a long study, it will be difficult to maintain adherence, and participants not in Group E will have less motivation to set goals independently and complete their e-logbooks during the retention phase after the first 6 weeks. Videos may serve as a reminder of how to optimally perform the interventions so participants will be encouraged and have a desire to continue with the intervention. Those who are in Group E will receive reminders to set goals and complete their e-logbooks, and periodic online questionnaires could also help remind all participants to continue with the intervention. Seasonal challenges could make it more difficult for participants in Canada to remain self-motivated, so adjustments in the commencement of the study to avoid the winter months (ie, starting at the end of March and continuing until late September) is a potential solution.

After the completion of this RCT, the People Getting a Grip on Rheumatoid Arthritis (PGrip-RA) program on the arthritis.ca website can be disseminated, for instance, through the Facebook page of The Arthritis Society (Canada) as well as that of Arthritis and Osteoporosis (Australia) for a broader group of arthritic individuals, especially for use in rural or underserved areas The use of social media as a method to disseminate self-management programs is novel and has a high potential to be a method to increase access to information for individuals with arthritis, particularly in rural or underserved areas.

## Multimedia Appendix 1

CONSORT-EHEALTH checklist V1.6.2 [40].

## Abbreviations

AHPA: Arthritis Health Profession Association
ANOVA: analysis of variance
ASES: Stanford Arthritis Self-Efficacy Scale
EQ-5D-5L: EuroQoL Index
HMESE: Hypothesized Model of Effects of Self-Efficacy-Enhancing Interventions for People With Chronic Diseases
ICT: information communication technologies
ID: identification number
KT: knowledge translation
KTA: knowledge-to-action
MRC: Medical Research Council
OA: osteoarthritis
PA: physical activity
PAR: physical activity readiness
PAR-Q: Physical Activity Readiness Questionnaire
PGrip: People Getting a Grip on Arthritis
QALY: quality-adjusted life year
QOL: quality of life
RA: rheumatoid arthritis
RCT: randomized control trial
SMP: self-management program
SUS: System Usability Scale
TAM: Technology Acceptance Model
TAS: The Arthritis Society
TENS: transcutaneous electrical nerve stimulation

## References

[1] McIlhenny CV, Guzic BL, Knee DR, Wendekier CM, Demuth BR, Roberts JB (2011). Using technology to deliver healthcare education to rural patients. Rural Remote Health.

[2] Li JS, Barnett TA, Goodman E, Wasserman RC, Kemper AR, American Heart Association Atherosclerosis‚ Hypertension and Obesity in the Young Committee of the Council on Cardiovascular Disease in the Young‚ Council on EpidemiologyPrevention‚Council on Nutrition‚ Physical ActivityMetabolism (2013). Approaches to the prevention and management of childhood obesity: the role of social networks and the use of social media and related electronic technologies: a scientific statement from the American Heart Association. Circulation.

[3] (2013). A problem worth solving. The rising cost of musculoskeletal conditions in Australia.

[4] Arthritis Alliance of Canada (2011). The Impact of Arthritis in Canada: Today and Over the Next 30 Years.

[5] Badley EM, Davis AM (2012). Meeting the challenge of the ageing of the population: issues in access to specialist care for arthritis. Best Pract Res Clin Rheumatol.

[6] Shigaki CL, Smarr KL, Siva C, Ge B, Musser D, Johnson R (2013). RAHelp: an online intervention for individuals with rheumatoid arthritis. Arthritis Care Res (Hoboken).

[7] Riemsma RP, Kirwan JR, Taal E, Rasker JJ (2003). Patient education for adults with rheumatoid arthritis. Cochrane Database Syst Rev.

[8] Brosseau L, Wells G, Tugwell P, Egan M, Dubouloz CJ, Welch VA, Trafford L, Sredic D, Pohran K, Smoljanic J, Vukosavljevic I, De Angelis G, Loew L, McEwan J, Bell EEM, Finestone HM, Lineker S, King J, Jelly W, Casimiro L, Haines-Wangda A, Russell-Doreleyers M, Laferriere L, Lambert K (2011). Ottawa Panel evidence-based clinical practice guidelines for patient education in the management of Rheumatoid Arthritis (RA). Health Education Journal.

[9] Davies CA, Spence JC, Vandelanotte C, Caperchione CM, Mummery WK (2012). Meta-analysis of internet-delivered interventions to increase physical activity levels. Int J Behav Nutr Phys Act.

[10] Murray E, Burns J, See TS, Lai R, Nazareth I (2005). Interactive Health Communication Applications for people with chronic disease. Cochrane Database Syst Rev.

[11] Wicks P, Stamford J, Grootenhuis MA, Haverman L, Ahmed S (2014). Innovations in e-health. Qual Life Res.

[12] Eysenbach G (2001). What is e-health?. J Med Internet Res.

[13] Maher CA, Lewis LK, Ferrar K, Marshall S, De Bourdeaudhuij I, Vandelanotte C (2014). Are health behavior change interventions that use online social networks effective? A systematic review. J Med Internet Res.

[14] Brosseau L, Wells GA, Brooks S, De Angelis G, Bell M, Egan M, Poitras S, King J, Casimiro L, Loew L, Novikov M (2013). People getting a grip on arthritis II: An innovative strategy to implement clinical practice guidelines for rheumatoid arthritis and osteoarthritis patients through Facebook. Health Education Journal.

[15] Kind T, Huang ZJ, Farr D, Pomerantz KL (2005). Internet and computer access and use for health information in an underserved community. Ambul Pediatr.

[16] Vance K, Howe W, Dellavalle RP (2009). Social internet sites as a source of public health information. Dermatol Clin.

[17] Hamm MP, Chisholm A, Shulhan J, Milne A, Scott SD, Given LM, Hartling L (2013). Social media use among patients and caregivers: a scoping review. BMJ Open.

[18] Hamm MP, Chisholm A, Shulhan J, Milne A, Scott SD, Klassen TP, Hartling L (2013). Social media use by health care professionals and trainees: a scoping review. Acad Med.

[19] Coulter A, Ellins J (2007). Effectiveness of strategies for informing, educating, and involving patients. BMJ.

[20] Brady TJ (2012). Cost implications of self-management education intervention programmes in arthritis. Best Pract Res Clin Rheumatol.

[21] Napolitano MA, Hayes S, Bennett GG, Ives AK, Foster GD (2013). Using Facebook and text messaging to deliver a weight loss program to college students. Obesity (Silver Spring).

[22] Pereyra-Elías R, Mayta-Tristán P (2012). Recruiting researchers through Facebook. Epidemiology.

[23] Farmer AD, Bruckner Holt CE, Cook MJ, Hearing SD (2009). Social networking sites: a novel portal for communication. Postgrad Med J.

[24] Cain J, Policastri A (2011). Using Facebook as an informal learning environment. Am J Pharm Educ.

[25] Patrick K, Marshall SJ, Davila EP, Kolodziejczyk JK, Fowler JH, Calfas KJ, Huang JS, Rock CL, Griswold WG, Gupta A, Merchant G, Norman GJ, Raab F, Donohue MC, Fogg BJ, Robinson TN (2014). Design and implementation of a randomized controlled social and mobile weight loss trial for young adults (project SMART). Contemp Clin Trials.

[26] Cavallo DN, Tate DF, Ries AV, Brown JD, DeVellis RF, Ammerman AS (2012). A social media-based physical activity intervention: a randomized controlled trial. Am J Prev Med.

[27] Cobb NK, Jacobs MA, Saul J, Wileyto EP, Graham AL (2014). Diffusion of an evidence-based smoking cessation intervention through Facebook: a randomised controlled trial study protocol. BMJ Open.

[28] Bull SS, Levine DK, Black SR, Schmiege SJ, Santelli J (2012). Social media-delivered sexual health intervention: a cluster randomized controlled trial. Am J Prev Med.

[29] Côté J, Godin G, Guéhéneuc YG, Rouleau G, Ramirez-Garcìa P, Otis J, Tremblay C, Fadel G (2012). Evaluation of a real-time virtual intervention to empower persons living with HIV to use therapy self-management: study protocol for an online randomized controlled trial. Trials.

[30] Valle CG, Tate DF, Mayer DK, Allicock M, Cai J (2013). A randomized trial of a Facebook-based physical activity intervention for young adult cancer survivors. J Cancer Surviv.

[31] Bossen D, Buskermolen M, Veenhof C, de Bakker D, Dekker J (2013). Adherence to a web-based physical activity intervention for patients with knee and/or hip osteoarthritis: a mixed method study. J Med Internet Res.

[32] Bossen D, Veenhof C, Dekker J, de Bakker D (2013). The usability and preliminary effectiveness of a web-based physical activity intervention in patients with knee and/or hip osteoarthritis. BMC Med Inform Decis Mak.

[33] Bossen D, Veenhof C, Van Beek KE, Spreeuwenberg PM, Dekker J, De Bakker DH (2013). Effectiveness of a web-based physical activity intervention in patients with knee and/or hip osteoarthritis: randomized controlled trial. J Med Internet Res.

[34] Brosseau L, Wells G, Tugwell P, Egan M, Dubouloz CJ, Casimiro L, Robinson VA, Pelland L, McGowan J, Bell M, Finestone HM, Légaré F, Caron C, Lineker S, Haines-Wangda A, Russell-Doreleyers M, Hall M, Cedar P, Lamb M (2004). Ottawa Panel evidence-based clinical practice guidelines for therapeutic exercises and manual therapy in the treatment of rheumatoid arthritis in adults. Phys Ther.

[35] Brosseau L, Wells G, Tugwell P, Egan M, Dubouloz CJ, Casimiro L, Robinson VA, Pelland L, McGowan J, Bell M, Finestone HM, Légaré F, Caron C, Lineker S, Haines-Wangda A, Russell-Doreleyers M, Hall M, Cedar P, Lamb M (2004). Ottawa Panel evidence-based clinical practice guidelines for electrotherapy and thermotherapy interventions in the treatment of rheumatoid arthritis in adults. Phys Ther.

[36] Brosseau L, Wells GA, Tugwell P, Egan M, Dubouloz C, Welch VA, Trafford L, Sredic D, De Angelis G, King J, Jelly W, Casimiro L (2010). Ottawa Panel evidence-based clinical practice guidelines for patient education in the management of osteoarthritis. Health Education Journal.

[37] Lorig KR, Ritter PL, Laurent DD, Plant K (2008). The internet-based arthritis self-management program: a one-year randomized trial for patients with arthritis or fibromyalgia. Arthritis Rheum.

[38] People getting a grip on arthritis videos.

[39] Straus SE, Tetroe J, Graham ID (2013). Knowledge translation in health care: moving from evidence to practice. Edition.

[40] Eysenbach G, CONSORT-EHEALTH Group (2011). CONSORT-EHEALTH: improving and standardizing evaluation reports of Web-based and mobile health interventions. J Med Internet Res.

[41] Smith DH, Neutel JM, Lacourcière Y, Kempthorne-Rawson J (2003). Prospective, randomized, open-label, blinded-endpoint (PROBE) designed trials yield the same results as double-blind, placebo-controlled trials with respect to ABPM measurements. J Hypertens.

[42] Craig P, Dieppe P, Macintyre S, Michie S, Nazareth I, Petticrew M (2013). Developing and evaluating complex interventions: the new Medical Research Council guidance. Int J Nurs Stud.

[43] PGRIP2: Community page about arthritis.

[44] Adams R (1999). Revised Physical Activity Readiness Questionnaire. Can Fam Physician.

[45] Hoffmann TC, Glasziou PP, Boutron I, Milne R, Perera R, Moher D, Altman DG, Barbour V, Macdonald H, Johnston M, Lamb SE, Dixon-Woods M, McCulloch P, Wyatt JC, Chan AW, Michie S (2014). Better reporting of interventions: template for intervention description and replication (TIDieR) checklist and guide. BMJ.

[46] MacKay C, Veinot P, Badley E (2006). An overview of developments in comprehensive interdisciplinary models of care for arthritis: Provider and patient perspectives. Arthritis Community Research & Evaluation Unit (ACREU).

[47] Hanly JG, Canadian Council of Academic Rheumatologists (2001). Manpower in Canadian academic rheumatology units: current status and future trends. Canadian Council of Academic Rheumatologists. J Rheumatol.

[48] Brosseau L, Rahman P, Toupin-April K, Poitras S, King J, De Angelis G, Loew L, Casimiro L, Paterson G, McEwan J (2014). A systematic critical appraisal for non-pharmacological management of osteoarthritis using the appraisal of guidelines research and evaluation II instrument. PLoS One.

[49] Brosseau L, Rahman P, Poitras S, Toupin-April K, Paterson G, Smith C, King J, Casimiro L, De Angelis G, Loew L, Cavallo S, Ewan JM (2014). A systematic critical appraisal of non-pharmacological management of rheumatoid arthritis with appraisal of guidelines for research and evaluation II. PLoS One.

[50] Neville LM, O'Hara B, Milat A (2009). Computer-tailored physical activity behavior change interventions targeting adults: a systematic review. Int J Behav Nutr Phys Act.

[51] Norman GJ, Zabinski MF, Adams MA, Rosenberg DE, Yaroch AL, Atienza AA (2007). A review of eHealth interventions for physical activity and dietary behavior change. Am J Prev Med.

[52] Vandelanotte C, Spathonis KM, Eakin EG, Owen N (2007). Website-delivered physical activity interventions a review of the literature. Am J Prev Med.

[53] Sallis JF, Haskell WL, Wood PD, Fortmann SP, Rogers T, Blair SN, Paffenbarger RS (1985). Physical activity assessment methodology in the Five-City Project. Am J Epidemiol.

[54] Thompson A (2011). Rheumatoid arthritis – know your options.

[55] The Arthritis Society (2009). Physical activity & arthritis.

[56] Ory MG, Ahn S, Jiang L, Lorig K, Ritter P, Laurent DD, Whitelaw N, Smith ML (2013). National study of chronic disease self-management: six-month outcome findings. J Aging Health.

[57] Lorig K, Ritter PL, Plant K, Laurent DD, Kelly P, Rowe S (2013). The South Australia health chronic disease self-management Internet trial. Health Educ Behav.

[58] Goeppinger J, Lorig KR, Ritter PL, Mutatkar S, Villa F, Gizlice Z (2009). Mail-delivered arthritis self-management tool kit: a randomized trial and longitudinal followup. Arthritis Rheum.

[59] Marks R, Allegrante JP, Lorig K (2005). A review and synthesis of research evidence for self-efficacy-enhancing interventions for reducing chronic disability: implications for health education practice (part II). Health Promot Pract.

[60] Lorig K, Chastain RL, Ung E, Shoor S, Holman HR (1989). Development and evaluation of a scale to measure perceived self-efficacy in people with arthritis. Arthritis Rheum.

[61] Rogers E (1995). Diffusion of innovations.

[62] Davis FD, Bagozzi RP, Warshaw PR (1989). User Acceptance of Computer Technology: A Comparison of Two Theoretical Models. Management Science.

[63] Rabin R, de Charro F (2001). EQ-5D: a measure of health status from the EuroQol Group. Ann Med.

[64] Stolee P, Rockwood K, Fox RA, Streiner DL (1992). The use of goal attainment scaling in a geriatric care setting. J Am Geriatr Soc.

[65] Brosseau L, Wells GA, Kenny GP, Reid R, Maetzel A, Tugwell P, Huijbregts M, McCullough C, De Angelis G, Chen L (2012). The implementation of a community-based aerobic walking program for mild to moderate knee osteoarthritis (OA): a knowledge translation (KT) randomized controlled trial (RCT): part I: the uptake of the Ottawa Panel clinical practice guidelines (CPGs). BMC Public Health.

[66] Ellison NB, Steinfıeld C, Lampe C (2007). The benefıts of Facebook “friends”: social capital and college students’ use of online social network sites. J Comput Mediat Commun.

[67] Björk M, Gerdle B, Thyberg I, Peolsson M (2008). Multivariate relationships between pain intensity and other aspects of health in rheumatoid arthritis--cross sectional and five year longitudinal analyses (the Swedish TIRA project). Disabil Rehabil.

[68] Saturo J (2011). Measuring usability with the system usability scale (SUS).

[69] Venkatesh V, Davis FD (2000). A theoretical extension of the technology acceptance model: Four longitudinal field studies. Management Science.

[70] Maetzel A, Li LC, Pencharz J, Tomlinson G, Bombardier C, Community Hypertension and Arthritis Project Study Team (2004). The economic burden associated with osteoarthritis, rheumatoid arthritis, and hypertension: a comparative study. Ann Rheum Dis.

[71] Manca A, Hawkins N, Sculpher MJ (2005). Estimating mean QALYs in trial-based cost-effectiveness analysis: the importance of controlling for baseline utility. Health Econ.

[72] Bandura A, Adams NE, Hardy AB, Howells GN (1980). Tests of the generality of self-efficacy theory. Cogn Ther Res.

[73] Brosseau L, Lineker S, Bell M, Wells G, Casimiro L, Egan M, Cranney A, Tugwell P, Wilson KG, De Angelis G, Loew L (2010). People getting a grip on arthritis: A knowledge transfer strategy to empower patients with rheumatoid arthritis and osteoarthritis. Health Education Journal.

[74] Hayden-Wade HA, Coleman KJ, Sallis JF, Armstrong C (2003). Validation of the telephone and in-person interview versions of the 7-day PAR. Med Sci Sports Exerc.

[75] Eysenbach G (2004). Improving the quality of Web surveys: the Checklist for Reporting Results of Internet E-Surveys (CHERRIES). J Med Internet Res.

[76] Creswell JW (2013). Qualitative Inquiry and Research Design: Choosing Among Five Approaches.

[77] Cohen J (1988). The analysis of variance. Statistical power analysis for the behavioral sciences.

[78] Ontario Ministry of Health and Long-Term Care (2013). Ontario Case Costing Initiative.

[79] Ontario Ministry of Health and Long-Term Care (2014). Ontario health insurance (OHIP) schedule of benefits and fees.

[80] Ontario Ministry of Health and Long-Term Care (2010). Schedule of benefits for laboratory services. Toronto: Queen’s Printer for Ontario.

[81] Chaudhary MA, Stearns SC (1996). Estimating confidence intervals for cost-effectiveness ratios: an example from a randomized trial. Stat Med.

[82] Deyo RA, Walsh NE, Schoenfeld LS, Ramamurthy S (1990). Can trials of physical treatments be blinded? The example of transcutaneous electrical nerve stimulation for chronic pain. Am J Phys Med Rehabil.

