# Supplementary material for: Internet-Based Implementation of Non-Pharmacological Interventions of the "People Getting a Grip on Arthritis" Educational Program: An International Online Knowledge Translation Randomized Controlled Trial Design Protocol
Source: JMIR Res Protoc. 2015 Feb 3;4(1):e19. doi: 10.2196/resprot.3572 (PMC4342636; doi:10.2196/resprot.3572)
Supplement: Supplementary file 1 [file resprot_v4i1e19_app1.pdf]

CONSORT-EHEALTH Checklist V1.6.2 Report

(based on CONSORT-EHEALTH V1.6), available at [http://tinyurl.com/consort-ehealth-v1-6].

3572

Date completed

5/29/2014 17:21:42

by

Lucie Brosseau

"The implementation of effective non-pharmacological interventions of the People Getting a Grip educational program for individuals with rheumatoid arthritis through Internet-based delivery methods: An International Online Knowledge Translation Randomized Controlled Trial Design Protocol"

TITLE

1a-i) Identify the mode of delivery in the title

The following is an except from the manuscript title: "through Internet-based delivery methods".

1a-ii) Non-web-based components or important co-interventions in title

There were no non-web-based components. The interventions is mentioned in the title: "effective non-pharmacological interventions of the People Getting a Grip educational program".

1a-iii) Primary condition or target group in the title

The following is an excerpt from the manuscript title: "educational program for individuals with rheumatoid arthritis".

ABSTRACT

1b-i) Key features/functionalities/components of the intervention and comparator in the METHODS section of the ABSTRACT

"This KT randomized controlled trial (RCT) will use a Prospective Randomized Open-label Blinded-Endpoint (PROBE) design to compare four different intervention approaches of the PGrip-RA program to a control group only receiving electronic educational pamphlets about general self-management in RA via e-mail. Depending on group allocation, links to the Arthritis Society PGrip-RA material will be provided either through Facebook or by e-mail; one group will receive feedback online from trained health professionals…Participants will have access to the Internet-based material after the completion of the baseline questionnaires until the final follow-up questionnaire at 6 months. We will invite 200 patients from Canadian and Australian arthritis consumer associations to participate using online recruitment".

1b-ii) Level of human involvement in the METHODS section of the ABSTRACT

The abstract mentions that "one group will receive feedback online from trained health professionals". The other Facebook group will have the potential of participants interacting with each other, but not with health professionals. The e-mail groups will have no interaction with others, except for reminder e-mails to complete online questionnaires. Note that the human involvement is Internet-based rather than in-person.

1b-iii) Open vs. closed, web-based (self-assessment) vs. face-to-face assessments in the METHODS section of the ABSTRACT

"We will invite 200 patients from Canadian and Australian arthritis consumer associations to participate using online recruitment" at the email address PGrip@gmail.com." Outcomes are self-assessed because "Participants will have access to the Internet-based material after the completion of the baseline questionnaires until the final follow-up questionnaire at 6 months".

1b-iv) RESULTS section in abstract must contain use data

This criterion is not applicable to a protocol as there are no results at this point in time to report.

1b-v) CONCLUSIONS/DISCUSSION in abstract for negative trials

This criterion is not applicable to a protocol as there are no results at this point in time to report.

INTRODUCTION

2a-i) Problem and the type of system/solution

In terms of a stand-alone intervention: "People Getting a Grip on Arthritis (PGrip is an evidence-based educational program that is based on the Ottawa Panel guidelines (Brosseau et al., 2004a; Brosseau et al., 2004b; Brosseau et al., 2012a). It consists of education about numerous effective non-pharmacological self-management interventions for arthritis to improve health behavioural changes such as self-efficacy (Lorig et al., 2008). PGrip has been adapted by primary care providers and translated into lay words for patients to improve arthritis care in the community". For specific intended population and goals of the intervention: "Allowing patients with [rheumatoid arthritis] to have easy access to effective self-management programs will increase patient self-efficacy, optimize their quality of life (QOL), and reduce the burden on the limited number of health professionals in both Canadian and Australian health systems, especially in rural locations (McIlhenny 2011; Li 2013)" and "Current recommendations for future evaluation of the implementation of evidence-based self-management programs through online interventions (Hamm et al., 2013a & b; Coulter & Ellins, 2007; Brady, 2012; Napolitano et al., 2013; Shigaki et al., 2013) state the following have not been adequately evaluated: cost effectiveness, the comparative effectiveness of different online knowledge translation (KT) strategies, and which components of complex interventions provide the greatest benefit".

2a-ii) Scientific background, rationale: What is known about the (type of) system

"Although social media sites are attractive for disseminating public health messages, they remain underused by health professionals despite their low cost and wide reach (Vance et al., 2009). A recent systematic review (Maher et al., 2014) had nine of the 10 included studies considering the efficacy of interventions, such as online health social network websites (n=2), research health social network websites (n=3), and multi-component interventions delivered in part via pre-existing popular online social network websites (Facebook: n=4 and Twitter: n=1). This systematic review revealed significant improvements in outcomes related to health behavior change (effect sizes ranging from -0.05 (95% CI 0.45-0.35) to 0.84 (95% CI 0.49-1.19) (Maher et al., 2014). Facebook has also been shown to be a successful tool for recruiting and communicating with a research team, even in a multinational context (Pereyra-Elias et al., 2012); it provides a readily accessible portal for patients and healthcare professionals to share their experiences of investigation, diagnosis and management of disease (Farmer et al.,2009). In addition, Facebook has been the medium for a learning strategy, which included external experts and thought leaders, providing professional communication via social media (Cain et al., 2011). Facebook has not been used to deliver an effective self-management strategy in arthritis according to existing published protocols and studies using social media and ICT as a KT strategy (Patrick et 2014; Napolitano et al, 2012; Cavallo et al., 2012; Cobb et al, 2014; Bull et al., 2012; Côté et al., 2012; Valle et al., 2013; Bossen et al., 2013a, b, c). This complex RCT will identify which component of various patient education approaches delivered through different ICT methods is an important catalyst for stronger effect sizes and sustainable results compared to the control condition. People Getting a Grip on Arthritis (PGrip) is an evidence-based educational program (PGrip2: Community page about arthritis, n.d.) that is based on the Ottawa Panel guidelines (Brosseau et al., 2004a; Brosseau et al., 2004b; Brosseau et al., 2012a). It consists of education about numerous effective non-pharmacological self-management interventions for arthritis to improve health behavioural changes such as self-efficacy (Lorig et al., 2008). PGrip has been adapted by primary care providers and translated into lay words for patients to improve arthritis care in the community. The proposed PGrip-RA program will provide updated material. For the purpose of the proposed study, the video material will be made available to participants by providing them with a direct Uniform Resource Locator (URL) link to the Facebook webpage and/or The Arthritis Society (TAS) PGrip webpage via e-mail (The Arthritis Society, n.d.). The proposed RCT will examine the effectiveness of Facebook as a KT strategy to deliver effective self-management interventions (with or without the participation of health professionals). The protocol builds on a pilot project by proposing a larger-scale RCT that involves health professionals and electronic dissemination of the self-management guidelines and broadening the study to include an international site".

METHODS

3a) CONSORT: Description of trial design (such as parallel, factorial) including allocation ratio

"The general hypothesis is that an online program provided to patients with RA using Facebook in combination with arthritis health professional support and electronic educational pamphlets can serve as a useful and economical method for KT. The objective of the first dimension is to examine the effect of the use of information communication technologies (ICT) (ie, Facebook and e-mails) as KT strategies to implement the evidence-based PGrip-RA self-management educational program. The objective of the second dimension is to examine the effect of the implementation of the PGrip-RA program on clinical outcomes".

3b) CONSORT: Important changes to methods after trial commencement (such as eligibility criteria), with reasons

Changes made to the methods is not applicable as the manuscript is a protocol.

3b-i) Bug fixes, Downtimes, Content Changes

Changes made to the methods is not applicable as the manuscript is a protocol.

4a) CONSORT: Eligibility criteria for participants

"Participants must fulfill the following criteria: 1) be between 18-75 years old; 2) be diagnosed with RA; 3) reside in Canada or Australia; 4) be without serious co-morbidities or chronic disease (eg, cancer or other illness) judged by the patient or study physician to make participation in this study inadvisable; 5) use medication that is not expected to change during the study period; 6) be self-reported as inactive (30 minutes of moderate physical activity, 5 times or less per week) or not using physical interventions or agents other than prescribed medication; 7) be without concurrent face-to-face consultation with a health care provider other than general practitioners or rheumatologists for RA for the recruitment period and the duration of the study; 8) be capable of using and accessing the Internet weekly and a functioning e-mail account during the study duration (6 months); 9) be free from contraindications to exercise without supervision established by the revised version of the physical activity (PA) questionnaire [42]; 10) be able to communicate in English; 11) be a new participant (ie, not having participated in either of the two previous PGrip pilot studies); and 12) be willing to sign informed consent."

4a-i) Computer / Internet literacy

Since the recruitment will be online, the participants will need to be computer literate: "Participants must fulfill the following criteria…be capable of using and accessing the Internet weekly and a functioning e-mail account during the study duration (6 months)".

4a-ii) Open vs. closed, web-based vs. face-to-face assessments:

"Recruitment methods include an advertisement on the Facebook page of The Arthritis Society, arthritis patient associations' electronic newsletter websites and other health-related websites. Potential participants will register by e-mail and will be invited to complete an online eligibility/admission questionnaire to ensure that they meet the study's selection criteria prior to randomization". The trial is purely Internet-based. "Participants will have the opportunity to share their unique perspective on living with arthritis and how they plan to integrate the effective self-management interventions into their daily lives by posting comments on the "wall" of the Facebook group page". This section indicates that those in the Facebook groups will not be able to maintain anonymity within their study group because their names will appear in the group members list and when they post comments on the wall. However, those with e-mail will be quasi anonymous because they will not know who the other members of the group are. The consent form will describe this specific information to the participants. All emails will use a Bcc to maintain confidentiality. Facebook accounts require a person's information and every time a participant completes an online survey they will be asked for their address (as mentioned in the consent form) to send the $30 gift card. These steps will help reduce the risk of participants having multiple identities.

4a-iii) Information giving during recruitment

"An online invitation letter with informed consent will be sent to the eligible study participants by e-mail. Once informed consent is obtained, participants will be invited to complete an online baseline questionnaire".

4b) CONSORT: Settings and locations where the data were collected

Data collection has not yet taken place since this is a protocol. It will be collected online using the Fluid Survey online questionnaires at baseline, 6 weeks, and 3- and 6-months follow-up, and metrics will be taken from Facebook and the video counts.

4b-i) Report if outcomes were (self-)assessed through online questionnaires

Outcomes were not self-assessed, but they are self-reported, since participants will be filling out online questionnaires via Fluid Survey at baseline, 6 weeks, and 3- and 6-months follow-up. The manuscript indicates that "A blinded independent assessor will be trained to assess the online self-reported questionnaires".

4b-ii) Report how institutional affiliations are displayed

In the online consent form all study investigators' names and institutional affiliations will be stated.

5) CONSORT: Describe the interventions for each group with sufficient details to allow replication, including how and when they were actually administered

5-i) Mention names, credential, affiliations of the developers, sponsors, and owners

The People Getting a Grip on arthritis educational program was developed by the following authors: Lucie Brosseau, Sydney Brooks, Mary Bell, George A. Wells, Lynn Casimiro, Mary Egan, Ann Cranney, Peter Tugwell, Keith G. Wilson.

5-ii) Describe the history/development process

"The following key elements from the MRC framework been accomplished: development through PGrip based on the Ottawa Panel guidelines; and feasibility and piloting, with the conduction of a previous pilot study". With the conduction of the pilot study

5-iii) Revisions and updating

This criterion is not applicable to a protocol as the study has yet to be conducted.

5-iv) Quality assurance methods

A $30 gift card for completing online questionnaires will serve as an incentive for participants to provide accurate information.

5-v) Ensure replicability by publishing the source code, and/or providing screenshots/screen-capture video, and/or providing flowcharts of the algorithms used

The websites to be used are cited and the URL links (as well as Webcitations) are included in the references of the manuscript. The flowcharts of the algorithms are not possible to include at present since the study has not yet commenced.

5-vi) Digital preservation

The Facebook page to be used failed to be archived by webcite. However, all other webpages presented the URL and the archived webcite link in the references.

5-vii) Access

"After randomization, the participant will be informed through e-mail of their group assignment. Participants in the interventions groups (Groups E and D) will receive specific confidential information for log-in purposes". For those in the Facebook intervention groups, they require a previously existing Facebook account or must create one. Participants did not have to pay any fees, but they "will receive a C$30.00 gift certificate for each completed questionnaire and a personalized certificate of participation".

5-viii) Mode of delivery, features/functionalities/components of the intervention and comparator, and the theoretical framework

"There are two theoretically-based dimensions refining the KTA framework concepts for Monitoring Knowledge-Use and Evaluated Outcomes (Tables 2-4). The first dimension is to examine the effect of the implementation of the PGrip-RA program on clinical outcomes. The Hypothesized Model of Effects of Self-Efficacy-Enhancing Interventions for People with Chronic Diseases (HMESE) (Marks & Allegante, 2005) (Figure 3) adapted from the Self-Efficacy and Social Cognitive Theory developed by Bandura (1980) and by Lorig (1989) for arthritis and chronic disease assessment purposes. The second dimension is to examine the effect of the use of ICT, (i.e., Facebook and e-mails) as KT strategies to implement the evidence-based PGrip-RA educational program. This will be measured by the Diffusion of innovation model (Rogers 1995) and more specifically The Technology Acceptance Model (TAM) (Davis, 1989) (Figure 4)". The manuscript provided information about Facebook as the system to use for the study and was mentioned in item 2aii. Communication was asynchronous because each intervention group did not receive the same level of interaction; only the Facebook groups would be able to communicate with others by posting on the private group wall, while Group E could also communicate with various trained health professionals during the three 2-week modules (spanning 6 weeks in total). In addition, "For the purpose of the proposed study, the video material will be made available to participants by providing them with a direct Uniform Resource Locator (URL) link to the Facebook webpage and/or The Arthritis Society (TAS) PGrip webpage via e-mail (The Arthritis Society, n.d.)".

5-ix) Describe use parameters

"Participants will take part in three separate self-management online modules, each over the course of two weeks" for a total of six weeks. As for instructions provided to the users, "Similar to the PGrip pilot study (Brosseau et al., 2014a; PGrip: community page about arthritis, n.d.), video presentations will include narrated PowerPoint presentations with simplified, concise instructions on how to perform/apply each self-management intervention and case studies illustrating their appropriateness and relevance. In addition, video presentations of practical sessions including a health professional describing step-by-step instructions while performing the evidence-based intervention will also be posted on each Facebook group page".

5-x) Clarify the level of human involvement

"A group of three trained health professionals, representing three professions (physiotherapy, occupational therapy, and kinesiology) from the Arthritis Health Profession Association (AHPA) will be asked to read the comments and questions participants write to each other on the “wall” and will give feedback to the participants on a weekly basis… The health professionals will participate in a half-day workshop at the University of Ottawa prior to the study, where they will receive training and information on evidence-based practice and the selected self-management interventions (MacKay et al., 2006; Hanly et al., 2001; Brosseau et al., 2014b & c; Neville et al., 2009; Norman et al., 2007; Vandelanotte et al., 2007). Training will consist of Ottawa Panel guidelines, PGrip-RA material using PowerPoint presentations and videos, and frequently asked questions from the pilot study (Brosseau et al., 2014a)… During each 2 week module the respective health professional(s) will monitor the Facebook page on three separate days (Monday, Wednesday, and Friday for 4 hours each day), review all of the participants’ written comments and provide feedback". •Table 1 in the manuscript provides the details about when each health professional is involved in the study. Note that there is no face-to-face human involvement as this is an entirely Internet-based study. The health professionals will act more as patient educators in the RCT, whereas they provide direct care in their day-to-day practice.

5-xi) Report any prompts/reminders used

"Using the “wall” on the Facebook page for Groups E and D, our research team will provide updates and reminders to all participants regarding deadlines to complete questionnaires". In addition, "Goal setting will not be required for the participants in the four other groups. However, study participants in all groups will record their physical activities and participation in PGrip interventions using the 7-Day PAR calendar (Sallis et al., 1985)". • All participants will receive a reminder message by Facebook and/or email at baseline, 6 weeks, 3- and 6-months to complete the online questionnaires. They will also have the incentive of receiving a $30 gift card as mentioned above.

5-xii) Describe any co-interventions (incl. training/support)

"Figure 2 indicates that each group received an e-pamphlet from The Arthritis Society about general self-management in rheumatoid arthritis; this is provided for Groups D and E in addition to the Facebook intervention, but both are stand-alone interventions". •Due to the self-management programme in this study, the participants learn the interventions by themselves by watching the instructional videos. This does not differ from a real life scenario, thus it is generalizable. However, Group E receives support from health care professionals that is not generalizable to their everyday life.

6a) CONSORT: Completely defined pre-specified primary and secondary outcome measures, including how and when they were assessed

The primary outcome is self-efficacy. "Self-efficacy was chosen as the primary outcome, as the self-management interventions consist of various activities to ameliorate symptoms associated with RA, principally pain. The measurement of self-efficacy will therefore capture the effectiveness of all interventions regardless of the specific type of self-management strategy. The Stanford Arthritis Self-Efficacy Scale (ASES), a valid tool with an internal consistency reliability of 0.94 [57], will be used to assess participants' self-efficacy (Tables 2 and 4). The sub-scales of the ASES tool (self-efficacy to improve function and other symptoms) will be used for secondary outcomes. The internal consistency reliability of the pain scale is 0.75 with a test-retest reliability of 0.87, while the internal consistency reliability of the pain scale is 0.87 with a test-retest reliability of 0.90 [57]". "Knowledge acquisition will be measured by questionnaires used in the previous pilot study [12]. Participants' pre-program knowledge of the self-management interventions will be assessed at baseline, and post-program knowledge will be measured at 6 weeks immediate post intervention, and at 3- and 6-months follow-up (Tables 2 and 3). Participants will be asked to complete a series of questions using a Likert scale to determine which self-management strategy options are effective for treating RA. Also, a logbook, used in a previous RCT [62] will be filled out by each participant as the KTA framework step "monitoring the knowledge use" using the 7-Day PAR calendar and a bi-weekly questionnaire on potential changes in PA, medication intake, habits, and adverse events. Knowledge acquisition related to ICT use will also be performed". "Intention to use the PGrip-RA self-management interventions will be measured via questionnaires used in the previous pilot study (Tables 2 and 3) [12]. Study participants in all 5 groups will be asked to set goals bi-weekly regarding any self-management interventions offered by PGrip-RA by themselves (participants in groups A, B, C and D) or with a health professional (Group E)". "Actual use of the PGrip-RA self-management interventions will be measured by questionnaires used in the two pilot studies (Tables 2 and 4) [12,69]. The number of views of the YouTube videos and the number of comments and postings (Facebook or e-mails) will be recorded. Furthermore, the Facebook Intensity Scale will be used to measure participants' overall engagement in Facebook for groups E and D only [63]. PGrip-RA program adherence will be measured with the actual use questionnaire [12] and also by calculating the proportion of the number of intervention sessions performed divided by the number of sessions prescribed (eg, walking program 3 times a week as recommended in the Ottawa Panel guidelines [33,34]) and recorded in the participants' online logbooks. A logbook used in a previous RCT [62] will be filled out by each participant as the "monitoring the knowledge" phase of the KTA framework (Figure 1) using the validated 7-Day Physical Activity Readiness (PAR) calendar [51,70] and a bi-weekly questionnaire on potential changes in PA, medication intake, habits and adverse events. The calendar proposed by the 7-Day PAR [51] incorporated in the logbooks will be used as a self-report questionnaire to calculate the number of intervention sessions each participant will attend each week. The 7-Day PAR will also be adapted to record prescribed numbers of application sessions of other physical interventions (eg, physical activity, TENS, etc.) to be optimally effective according to the Ottawa Panel guidelines, [33,34]. Actual individual recordings in the 7-Day PAR calendar will be compared with PGrip-RA intervention recommendations using the long goal attainment scale [61]. The Long Term Goal Attainment Scaling is a validated tool that will measure participants' long term goal attainment levels (Tables 2 and 4). It includes five goal attainment levels: 1) -2 (much worse than expected), 2) -1 (somewhat less than expected), 3) 0 (expected level), 4) +1 (somewhat better than expected) and 5) +2 (much better than expected) [61]". "The self-efficacy function subscale of the ASES will be used to measure participants' self-efficacy to improve their functional status (Tables 2 and 4). The internal consistency reliability of this scale is 0.90 with a test-retest reliability of 0.85 (Tables 2 and 4) [57]". "Quality of life will be assessed using the "EuroQoL Index" (EQ-5D-5L) (Tables 2 and 4). It is the most commonly used and extensively validated measure of health-related quality of life [60]. It includes five domains: 1) mobility, 2) self-care, 3) usual activities, 4) pain/discomfort, and 5) anxiety/depression. The scoring system has 5 levels: no problems, slight problems, moderate problems, severe problems, and extreme problems [60]. The EQ-5D-5L is an integral component of the economic analysis detailed later (Tables 2 and 4)". "Participants in all groups will be assessed according to their level of usability with their respective ICT KT strategy (ie, Facebook or e-mail). The System Usability Scale (SUS) instrument (Tables 2 and 4), an empirically validated tool [65], as well as the adapted technology acceptance model (TAM) 2 scale [66], will be used to measure participants' usability perception at 6 weeks immediate post intervention, and 3- and 6-months follow-up (Tables 2 and 4)". "Study participants' self-reported assessment of pain intensity will be recorded on an online 100-millimeter (mm) visual analogue scale (VAS) (Tables 2 and 4), where 0 mm represents no pain and 100 mm maximal pain (Tables 2 and 4) [64]".

6a-i) Online questionnaires: describe if they were validated for online use and apply CHERRIES items to describe how the questionnaires were designed/deployed

The CHERRIES checklist is not applicable for this protocol because the online questionnaires have not been used to collect results at this point and the checklist is meant for reporting the results of an Internet survey. All scales used in the study are valid except for intent to use questionnaire and the Actual Use Questionnaire; their psychometrics have not been validated, but they are feasible based on the previous pilot study.

6a-ii) Describe whether and how "use" (including intensity of use/dosage) was defined/measured/monitored

"Actual use of the PGrip-RA self-management interventions will be measured by questionnaires used in the two pilot studies (Brosseau et al., 2012c; Brosseau et al., 2014a) (Tables 2 and 4). The number of views of the YouTube videos and the number of comments and postings (Facebook or e-mails) will be recorded. Furthermore, the Facebook Intensity Scale will be used to measure participants' overall engagement in Facebook for groups E and D only (Ellison et al., 2007). PGrip-RA program adherence will be measured with the actual use questionnaire (Brosseau et al., 2014a) and also by calculating the proportion of the number of intervention sessions performed divided by the number of sessions prescribed (eg, walking program 3 times a week as recommended in the Ottawa Panel guidelines, 2004a, b) and recorded in the participants' online logbooks. A logbook used in a previous RCT (Brosseau et al. 2012b) will be filled out by each participant as the "monitoring the knowledge" phase of the KTA framework (Figure 1) using the validated 7-Day Physical Activity Readiness (PAR) calendar (Sallis et al., 1985; Hayden-Wade et al., 2003) and a bi-weekly questionnaire on potential changes in PA, medication intake, habits and adverse events. The calendar proposed by the 7-Day PAR (Sallis et al., 1985) incorporated in the logbooks will be used as a self-report questionnaire to calculate the number of intervention sessions each participant will attend each week. The 7-Day PAR will also be adapted to record prescribed numbers of application sessions of other physical interventions (eg, physical activity, TENS, etc.) to be optimally effective according to the Ottawa Panel guidelines, 2004a, b. Actual individual recordings in the 7-Day PAR calendar will be compared with PGrip-RA intervention recommendations using the long goal attainment scale (Stolee et al., 1992). The Long Term Goal Attainment Scaling is a validated tool that will measure participants' long term goal attainment levels (Tables 2 and 4). It includes five goal attainment levels: 1) -2 (much worse than expected), 2) -1 (somewhat less than expected), 3) 0 (expected level), 4) +1 (somewhat better than expected) and 5) +2 (much better than expected) (Stolee et al., 1992)".

6a-iii) Describe whether, how, and when qualitative feedback from participants was obtained

As previously mentioned, feedback from participants will be received during the post-intervention and follow-up questionnaires.

6b) CONSORT: Any changes to trial outcomes after the trial commenced, with reasons

This is not applicable as the current protocol has no results as of yet.

7a) CONSORT: How sample size was determined

7a-i) Describe whether and how expected attrition was taken into account when calculating the sample size

"We anticipate similar compliance rates as in our pilot study (Brosseau et al., 2014a). Only 1% of participants did not complete the baseline questionnaire and 20% did not complete the final 3-month follow-up questionnaire. Based on data from our pilot study (Brosseau et al., 2014a), only two participants out of 99 dropped out of the study. Participants were considered dropouts if they indicated they no longer wanted to participate in the study. The sample size has been adjusted accordingly for the proposed study". Figure 5 presents the sample size formula used.

7b) CONSORT: When applicable, explanation of any interim analyses and stopping guidelines

After the baseline questionnaire the next analysis will take place at 6 weeks, immediately post intervention.

8a) CONSORT: Method used to generate the random allocation sequence

Allocation sequence was "based on a sequence of computer generated random numbers".

8b) CONSORT: Type of randomisation; details of any restriction (such as blocking and block size)

Use of "a blocking factor (randomly varying between 4 and 6)".

9) CONSORT: Mechanism used to implement the random allocation sequence (such as sequentially numbered containers), describing any steps taken to conceal the sequence until interventions were assigned

"The research assistant, who is not involved in data collection, will contact the research study Methods Centre data manager. Prior to running the randomization program, the data manager will document the participant's initials (first and last) and date of birth (month and year). After running the program, the data manager will document the intervention assignment with the participant information, assign a study identification number (ID) and then inform the research assistant of the assignment and participant ID. This process will help ensure concealment of allocation".

10) CONSORT: Who generated the random allocation sequence, who enrolled participants, and who assigned participants to interventions

"a.After the potential participant registers online at PGrip@gmail.com, they will be contacted by the research coordinator and their eligibility will be confirmed. If eligible and consenting, the participant will then be randomly allocated to one of the five study groups (Group A, B, C, D or E) using the central randomization scheme. The research assistant, who is not involved in data collection, will contact the research study Methods Centre data manager. Prior to running the randomization program, the data manager will document the participant's initials (first and last) and date of birth (month and year). After running the program, the data manager will document the intervention assignment with the participant information, assign a study identification number (ID) and then inform the research assistant of the assignment and participant ID. This process will help ensure concealment of allocation".

11a) CONSORT: Blinding - If done, who was blinded after assignment to interventions (for example, participants, care providers, those assessing outcomes) and how

11a-i) Specify who was blinded, and who wasn't

"Mentions that only the independent assessor is blinded and they are the one assessing the online questionnaires; the others involved in the study are unblinded and a reason is given to explain why; also mentions that it follows the PROBE study design: "the interventions, the participants, and the research coordinator administering the program will be unblended…A blinded independent assessor will be trained to assess the online self-reported questionnaires". "Investigators will be blinded to intervention assignment throughout the study period".

11a-ii) Discuss e.g., whether participants knew which intervention was the "intervention of interest" and which one was the "comparator"

All participants will know that each group received a general self-management e-pamphlet and that the PGrip-RA educational program is evidence-based.

11b) CONSORT: If relevant, description of the similarity of interventions

This item is not applicable to the protocol as there was no sham intervention used.

12a) CONSORT: Statistical methods used to compare groups for primary and secondary outcomes

"For the primary research questions, an analysis of variance (ANOVA) will be conducted to compare groups A-E on the primary outcome, self-efficacy to manage pain (ASES), at 6-months follow-up. In particular, Dunnett's multiparameter test will compare groups B-E individually to group A on the primary outcome. If clinically important differences in baseline variables are found, the interventions will be compared using multiple regression adjusting for these baseline variables and similar multiparameter tests will be conducted. For the secondary outcomes the same analysis strategy considered for the primary research question will be followed. Furthermore, the change from baseline to 3 months in the measures will be analyzed in a similar fashion (Textbox 1)".

12a-i) Imputation techniques to deal with attrition / missing values

"To account for a potential loss to follow-up, the sample size has been adjusted to accommodate a 20% loss which is typical of the losses in similar past studies; that is, 32⁄(1-.2)=40 per group, and in total 200". "Data analysis will be performed using SPSS 21 and will be conducted on an intention-to-treat basis. Descriptive statistics such as proportions, means and standard deviation will be used to summarize baseline variables across the five study groups (Groups A-E). Baseline characteristics will be assessed to ensure there are no differences among the study groups. For the primary research questions, an analysis of variance (ANOVA) will be conducted to compare groups A-E on the primary outcome, self-efficacy to manage pain (ASES), at 6-months follow-up. In particular, Dunnett's multiparameter test will compare groups B-E individually to group A on the primary outcome. If clinically important differences in baseline variables are found, the interventions will be compared using multiple regression adjusting for these baseline variables and similar multiparameter tests will be conducted. For the secondary outcomes the same analysis strategy considered for the primary research question will be followed. Furthermore, the change from baseline to 3 months in the measures will be analyzed in a similar fashion (Textbox 1). In order to assess the importance of the different components making up the interventions for Groups B-E, ANOVA will be conducted and a posterior test using Tukey's honestly significance difference will compare Group E to D, Group D to C, Group C to B and Group B to A. This analysis will be considered for all outcomes. In addition, the outcomes will be compared from baseline to 6 weeks immediate post-intervention, 3- and 6-months follow-up using a 2-way ANOVA with the between factor as the study groups (Groups A-E) and the within factor as time (baseline, 6 weeks, 3- and 6-months)".

12b) CONSORT: Methods for additional analyses, such as subgroup analyses and adjusted analyses

"Utility values derived from the EQ-5D-5L responses (Tables 2 and 4) will be used to estimate QALYs for the 6-month period adjusting for baseline utility. The economic analysis will compare the incremental cost per QALY gained by each intervention group (Groups B-E) compared to the control (Group A) at 6-months follow-up. In order to estimate and adjust for the uncertainty of the incremental cost and effectiveness, probabilistic analysis will be conducted using non-parametric bootstrapping (Chaudhary et al., 1996)".

RESULTS

13a) CONSORT: For each group, the numbers of participants who were randomly assigned, received intended treatment, and were analysed for the primary outcome

Participants flow is not applicable for this protocol as the study has not yet commenced.

13b) CONSORT: For each group, losses and exclusions after randomisation, together with reasons

Losses and exclusions after randomization are not applicable for this protocol as the study has not yet commenced.

13b-i) Attrition diagram

An attrition diagram is not applicable for this protocol as the study has not yet commenced.

14a) CONSORT: Dates defining the periods of recruitment and follow-up

No specific dates have been set, although the study is anticipated to commence in the fall of 2014. The study length is 6 weeks with 3- and 6-months follow-up. As for recruitment, the previous pilot study recruited around 100 participants in under 1 month. This will be used as a guideline for the recruitment timeline of this study.

14a-i) Indicate if critical "secular events" fell into the study period

Secular events are not applicable for this protocol as the study has not yet commenced.

14b) CONSORT: Why the trial ended or was stopped (early)

This is not applicable for the protocol as the study has not yet commenced.

15) CONSORT: A table showing baseline demographic and clinical characteristics for each group

This is not applicable for the protocol as the study has not yet commenced.

15-i) Report demographics associated with digital divide issues

This is not applicable for the protocol as the study has not yet commenced.

16a) CONSORT: For each group, number of participants (denominator) included in each analysis and whether the analysis was by original assigned groups

16-i) Report multiple "denominators" and provide definitions

This is not applicable for the protocol as the study has not yet commenced.

16-ii) Primary analysis should be intent-to-treat

The primary and secondary analyses will be intent-to-treat.

17a) CONSORT: For each primary and secondary outcome, results for each group, and the estimated effect size and its precision (such as 95% confidence interval)

This is not applicable for the protocol as the study has not yet commenced.

17a-i) Presentation of process outcomes such as metrics of use and intensity of use

See item 6aii for detailed information about the Facebook metrics as well as qualitative measures of counting the number of posts or comments in the group pages. "ii.“Self-efficacy is one’s belief and confidence to perform a given behaviour, such as exercise (Bandura et al., 1980; Lorig et al. 1989; Marks & Allegrante, 2005). Self-efficacy was chosen as the primary outcome, as the self-management interventions consist of various activities to ameliorate symptoms associated with RA, principally pain. The measurement of self-efficacy will therefore capture the effectiveness of all interventions regardless of the specific type of self-management strategy. The Stanford Arthritis Self-Efficacy Scale (ASES), a valid tool with an internal consistency reliability of 0.94 (Lorig et al., 1989), will be used to assess participants’ self-efficacy (Tables 2 and 4). The sub-scales of the ASES tool (self-efficacy to improve function and other symptoms) will be used for secondary outcomes. The internal consistency reliability of the pain scale is 0.75 with a test-retest reliability of 0.87, while the internal consistency reliability of the pain scale is 0.87 with a test-retest reliability of 0.90 (Lorig et al., 1989). Knowledge acquisition will be measured by questionnaires used in the previous pilot study (Brosseau et al., 2014a). Participants’ pre-program knowledge of the self-management interventions will be assessed at baseline, and post-program knowledge will be measured at 6 weeks immediate post intervention, and at 3- and 6-months follow-up (Tables 2 and 3). Participants will be asked to complete a series of questions using a Likert scale to determine which self-management strategy options are effective for treating RA. Also, a logbook, used in a previous RCT (Brosseau et al., 2012b) will be filled out by each participant as the KTA framework step “monitoring the knowledge use” using the 7-Day PAR calendar and a bi-weekly questionnaire on potential changes in PA, medication intake, habits, and adverse events. Knowledge acquisition related to ICT use will also be performed. Examples of how “knowledge use” and “intended use” were operationalized are presented in Table 3. Intention to use the PGrip-RA self-management interventions will be measured via questionnaires used in the previous pilot study (Brosseau et al., 2014a) (Tables 2 and 3). Study participants in all 5 groups will be asked to set goals bi-weekly regarding any self-management interventions offered by PGrip-RA by themselves (participants in groups A, B, C and D) or with a health professional (Group E). Actual use of the PGrip-RA self-management interventions will be measured by questionnaires used in the two pilot studies (Brosseau et al., 2012c – Pgrip authors; Brosseau et al., 2014a) (Tables 2 and 4). The number of views of the YouTube videos and the number of comments and postings (Facebook or e-mails) will be recorded. Furthermore, the Facebook Intensity Scale will be used to measure participants’ overall engagement in Facebook for groups E and D only (Ellison et al., 2007). PGrip-RA program adherence will be measured with the actual use questionnaire (Brosseau et al., 2014a) and also by calculating the proportion of the number of intervention sessions performed divided by the number of sessions prescribed (eg, walking program 3 times a week as recommended in the Ottawa Panel guidelines, 2004a, b) and recorded in the participants’ online logbooks. A logbook used in a previous RCT (Brosseau et al. 2012b) will be filled out by each participant as the “monitoring the knowledge” phase of the KTA framework (Figure 1) using the validated 7-Day Physical Activity Readiness (PAR) calendar (Sallis et al., 1985; Hayden-Wade et al., 2003) and a bi-weekly questionnaire on potential changes in PA, medication intake, habits and adverse events. The calendar proposed by the 7-Day PAR (Sallis et al., 1985) incorporated in the logbooks will be used as a self-report questionnaire to calculate the number of intervention sessions each participant will attend each week. The 7-Day PAR will also be adapted to record prescribed numbers of application sessions of other physical interventions (eg, physical activity, TENS, etc.) to be optimally effective according to the Ottawa Panel guidelines, 2004a, b. Actual individual recordings in the 7-Day PAR calendar will be compared with PGrip-RA intervention recommendations using the long goal attainment scale (Stolee et al., 1992). The Long Term Goal Attainment Scaling is a validated tool that will measure participants’ long term goal attainment levels (Tables 2 and 4). It includes five goal attainment levels: 1) -2 (much worse than expected), 2) -1 (somewhat less than expected), 3) 0 (expected level), 4) +1 (somewhat better than expected) and 5) +2 (much better than expected) (Stolee et al., 1992). The self-efficacy function subscale of the ASES will be used to measure participants’ self-efficacy to improve their functional status (Tables 2 and 4). The internal consistency reliability of this scale is 0.90 with a test-retest reliability of 0.85 (Lorig et al. 1989) (Tables 2 and 4). Quality of life will be assessed using the “EuroQoL Index” (EQ-5D-5L) (Tables 2 and 4). It is the most commonly used and extensively validated measure of health-related quality of life (Rabin R & de Charro F., 2001). It includes five domains: 1) mobility, 2) self-care, 3) usual activities, 4) pain/discomfort, and 5) anxiety/depression. The scoring system has 5 levels: no problems, slight problems, moderate problems, severe problems, and extreme problems (Rabin et al., 2001). The EQ-5D-5L is an integral component of the economic analysis detailed later (Tables 2 and 4). Participants in all groups will be assessed according to their level of usability with their respective ICT KT strategy (ie, Facebook or e-mail). The System Usability Scale (SUS) instrument (Tables 2 and 4), an empirically validated tool (Saturo, 2011), as well as the adapted technology acceptance model (TAM) 2 scale (Venkatesh & Davis, 2000), will be used to measure participants’ usability perception at 6 weeks immediate post intervention, and 3- and 6-months follow-up (Tables 2 and 4). Study participants’ self-reported assessment of pain intensity will be recorded on an online 100-millimeter (mm) visual analogue scale (VAS) (Tables 2 and 4), where 0 mm represents no pain and 100 mm maximal pain (Björk et al., 2008) (Tables 2 and 4)."

17b) CONSORT: For binary outcomes, presentation of both absolute and relative effect sizes is recommended

"A small to moderate effect size of 0.3 in pain self-efficacy was identified by the investigators and colleagues as being a minimal clinically important effect size to identify".

18) CONSORT: Results of any other analyses performed, including subgroup analyses and adjusted analyses, distinguishing pre-specified from exploratory

This is not applicable for this protocol as the study has not yet commenced.

18-i) Subgroup analysis of comparing only users

This is not applicable for this protocol as the study has not yet commenced.

19) CONSORT: All important harms or unintended effects in each group

This is not applicable for this protocol as the study has not yet commenced.

19-i) Include privacy breaches, technical problems

This is not applicable for this protocol as the study has not yet commenced.

19-ii) Include qualitative feedback from participants or observations from staff/researchers

This is not applicable for this protocol as the study has not yet commenced.

DISCUSSION

20) CONSORT: Trial limitations, addressing sources of potential bias, imprecision, multiplicity of analyses

20-i) Typical limitations in ehealth trials

"Blinding participants is impossible in this type of study, as is generally the case with physical rehabilitation RCTs (Deyo, 1990). We recognize that the results of this study will likely only be generalizable to RA patients who are computer literate and have Internet access. Furthermore, we are also aware of the potential bias of only recruiting patients online, though this has proven to be effective in the previous pilot study (Brosseau et al., 2014a). Another limitation involves the timeframe of the intervention, as they will not be assessed for adherence in the long-term beyond the 6-month follow-up. There is an increased risk of Type-1 error due to the presence of multiple outcomes (ie, multicollinearity)".

21) CONSORT: Generalisability (external validity, applicability) of the trial findings

21-i) Generalizability to other populations

"The results of this study will likely only be generalizable to RA patients who are computer literate and have Internet access".

21-ii) Discuss if there were elements in the RCT that would be different in a routine application setting
The manuscript mentions that prompts and reminders could be a challenge for people to be motivated to continue with goal-setting and this is not available outside of the study. More insight may come after the study is conducted.

22) CONSORT: Interpretation consistent with results, balancing benefits and harms, and considering other relevant evidence

22-i) Restate study questions and summarize the answers suggested by the data, starting with primary outcomes and process outcomes (use)
This is not applicable for this protocol as the study has not yet commenced.

22-ii) Highlight unanswered new questions, suggest future research
This is not applicable for this protocol as the study has not yet commenced.

Other information

23) CONSORT: Registration number and name of trial registry
"Australian New Zealand Clinical Trials Registry ACTRN12614000397617; http://www.anzctr.org.au/TrialSearch.aspx (Archived by WebCite at http://www.webcitation.org/6PrP0kQf8)".

24) CONSORT: Where the full trial protocol can be accessed, if available
The current manuscript is the protocol, thus this is not applicable.

25) CONSORT: Sources of funding and other support (such as supply of drugs), role of funders
"The authors are thankful to the Canadian Institute of Health Research (CIHR) for the funding obtained for the two pilot studies on Facebook conducted in Canada (CIHR # KTB-248028), as well as in Australia and for two planning grants (CIHR # KPE-290576 & CIHR # KPE- 201306PMH) to confirm the feasibility of the study and to develop the actual protocol".

X26-i) Comment on ethics committee approval
"Similar online methods were used in the previous pilot study [12] and this study was approved by the University of Ottawa (U of O) Ethics Committee (Certificate number: H11-12-10)".

x26-ii) Outline informed consent procedures
"An online invitation letter with informed consent will be sent to the eligible study participants by e-mail". The informed consent will describe the study groups, indicate the privacy levels for the Facebook groups and how comments will only be able to be seen by others within the same private group. In addition, author names and affiliations will be provided.

X26-iii) Safety and security procedures
The consent form will explain that the study will ensure privacy within the study, but that those in the private Facebook group will be able to see comments from others when posted.

X27-i) State the relation of the study team towards the system being evaluated
No conflicts of interest were declared.
